# Acoustic Correlates and Adult Perceptions of Distress in Infant Speech-Like Vocalizations and Cries

**DOI:** 10.3389/fpsyg.2019.01154

**Published:** 2019-05-29

**Authors:** Hyunjoo Yoo, Eugene H. Buder, Dale D. Bowman, Gavin M. Bidelman, D. Kimbrough Oller

**Affiliations:** ^1^Department of Communicative Disorders, The University of Alabama, Tuscaloosa, AL, United States; ^2^School of Communication Sciences and Disorders, The University of Memphis, Memphis, TN, United States; ^3^Institute for Intelligent Systems, The University of Memphis, Memphis, TN, United States; ^4^Department of Mathematical Sciences, The University of Memphis, Memphis, TN, United States; ^5^Department of Anatomy and Neurobiology, University of Tennessee Health Science Center, Memphis, TN, United States; ^6^Konrad Lorenz Institute for Evolution and Cognition Research, Klosterneuburg, Austria

**Keywords:** infant vocalizations, babbling, distress sounds, cry, adult perception, acoustic analysis, active perception, fuss

## Abstract

Prior research has not evaluated acoustic features contributing to perception of human infant vocal distress or lack thereof on a continuum. The present research evaluates perception of infant vocalizations along a continuum ranging from the most prototypical intensely distressful cry sounds (“wails”) to the most prototypical of infant sounds that typically express no distress (non-distress “vocants”). Wails are deemed little if at all related to speech while vocants are taken to be clear precursors to speech. We selected prototypical exemplars of utterances representing the whole continuum from 0 and 1 month-olds. In this initial study of the continuum, our goals are to determine (1) listener agreement on level of vocal distress across the continuum, (2) acoustic parameters predicting ratings of distress, (3) the extent to which individual listeners maintain or change their acoustic criteria for distress judgments across the study, (4) the extent to which different listeners use similar or different acoustic criteria to make judgments, and (5) the role of short-term experience among the listeners in judgments of infant vocalization distress. Results indicated that (1) both inter-rater and intra-rater listener agreement on degree of vocal distress was high, (2) the best predictors of vocal distress were number of vibratory regimes within utterances, utterance duration, spectral ratio (spectral concentration) in vibratory regimes within utterances, and mean pitch, (3) individual listeners significantly modified their acoustic criteria for distress judgments across the 10 trial blocks, (4) different listeners, while showing overall similarities in ratings of the 42 stimuli, also showed significant differences in acoustic criteria used in assigning the ratings of vocal distress, and (5) listeners who were both experienced and inexperienced in infant vocalizations coding showed high agreement in rating level of distress, but differed in the extent to which they relied on the different acoustic cues in making the ratings. The study provides clearer characterization of vocal distress expression in infants based on acoustic parameters and a new perspective on active adult perception of infant vocalizations. The results also highlight the importance of vibratory regime segmentation and analysis in acoustically based research on infant vocalizations and their perception.

## Introduction

### Overview of the Present Study

Early infant vocalizations are under investigation in the search for origins of language (Locke, 1993; Oller et al., 2016). The present research seeks to characterize foundations of language by focusing on infant vocalizations along a continuum ranging from the most prototypical of intensely distressful cry sounds (which we call wails) to the most prototypical of infant sounds that typically express no distress at all. These non-distress sounds are deemed to be specific precursors to speech (Stark et al., 1993; Locke, 2006; Kern et al., 2009; Molemans, 2011). In any research on the full range of vocal communication in human infants, a differentiation between cry sounds and speech-like vocalizations (hereafter “protophones” after Oller, 2000) is necessary, yet acoustic criteria for implementing the differentiation have not been provided. To that end, the present study aims to (1) assess the ability of adult human listeners to reliably (based on both inter-rater and intra-rater correlations) rate the distressfulness of a carefully chosen set of infant vocalizations representing the continuum of distress, and then (2) to determine acoustic predictors of the ratings of infant vocalizations in the first 2 months of life along the distress continuum. Additional goals are (3) to evaluate the extent to which individual listeners use the acoustic predictors in similar or different ways in their judgments of distress across multiple trial blocks (an intra-rater evaluation), and (4) to determine differences *across* listeners in how they use the acoustic parameters in their judgments (an inter-rater evaluation). Finally, we assessed (5) differences in how the listeners used the acoustic parameters based on whether they had had prior experience in coding of infant vocalizations. Goals 3–5 reflect interest in the evolutionary foundations of language, where Darwinian principles and tenets of evolutionary-developmental biology (evo-devo) predict variation and environmental sensitivity in the manifestations of evolvable traits (Müller and Newman, 2003; Bertossa, 2011). Our work will thus address the potentially fluid nature of perception of the early sounds of human communication, reflecting variation of the trait in the human population, and possibly reflecting active exploratory and adaptive perception (Gibson, 1979).

### Infant Speech-Like and Distress Vocalizations

Infants produce various vocalizations, including distress sounds and protophones. Child development researchers have often assumed that cry is predominant in the first months of life (Lester and Boukydis, 1992; Hoff, 2004; Várallyay and Benyó, 2007). Many have claimed that protophones develop *from* cries and thus appear to assume that protophones do not occur until 2–3 months of age (e.g., Takahashi et al., 2015). However, there is solid empirical evidence that human infants produce endogenous protophones from birth (Nathani et al., 2006; Yoo et al., 2014; Dominguez et al., 2016; Oller et al., 2016). Nathani et al. (2006) reported that protophones accounted for approximately two-thirds of all vocalizations at 0–2 months and >90% by 16–20 months. Dominguez et al. (2016) found infants at 2–4 days produced 2.7 protophones (that is, sounds that were neither vegetative, nor were the produced in a “cry state”) per minute, confirming that protophones are common from the first days of life.

To our knowledge, there has been no explicit dispute about whether the earliest vocalizations of infancy include protophones. Yet much research on vocalization in the first months has left the implication that protophones are either absent or insignificant at this early age because much research has focused on cries to the exclusion of protophones, providing no clear definitions distinguishing cries from other sounds and offering little if any mention of their existence (for reviews see Wasz-Höckert et al., 1985 or Oller, 2000). Even if researchers are to study protophones or distress sounds separately, distinguishing definitions are required.

Surprisingly, there appear to exist no studies providing explicit auditory or acoustic criteria for discriminating protophones from cries. Instead, two literatures have been pursued separately for decades with neither literature providing an auditory or acoustic explanation for how listeners have segregated the sounds. The cry literature has focused on analyses of cry sounds without offering acoustic criteria for differentiating these from protophones (Wolff, 1969; Michelsson et al., 1983, 1982; Wasz-Höckert et al., 1985; Green et al., 1987; Michelsson and Michelsson, 1999; LaGasse et al., 2005). Similarly, the protophone literature has focused analyses on non-cry sounds, without offering clear acoustic criteria for differentiating these from cry sounds (Oller, 1980; Koopmans-van Beinum and van der Stelt, 1986; Roug et al., 1989). It seems that researchers have mostly relied on situational elicitation criteria (e.g., sounds occurring immediately following a needle prick) when defining cry, and in the absence of immediate indicators suggesting pain or discomfort, they have assumed vocalizations were protophones, sometimes referring to these as “comfort” sounds (Stark et al., 1978). Thus, even though it is undeniable that humans must be able to identify infant sounds and differentiate them along the continuum from cry to protophones, science has as yet failed to determine the acoustic parameters that must be the source of the differentiation.

### Developing Criteria to Discriminate Protophones From Distress Vocalizations

Stark et al. (1993) investigated vocalizations from 51 infants birth to 18 months. In some regards the authors gave quite detailed descriptions of their coding system, but they did not indicate how fussing or cry vocalizations were differentiated from protophones or non-fussing sounds. Oller et al. (2013) investigated both infant protophones and fixed signals (i.e., cry and laugh) across the first year of life, conceding that for their vocal type coding “no definition was given [to the coders] for cry or laugh, since it was assumed that these terms would be applied appropriately without training” (p. 31 in the article’s Supporting Information Appendix). Fuller and Horii (1986) investigated fundamental frequency (f_0_), jitter, and shimmer in “pain cry,” “hunger cry,” “fussing,” and “cooing.” Again, however, no explicit auditory or acoustic criteria were provided to segregate sounds into the four types prior to analysis of f_0_, jitter, and shimmer. Instead, the authors defined vocal types based on the eliciting situation. In general, it appears that studies of cries and protophones have relied on intuitive or situational judgments by coders to differentiate infant vocal types. Such an approach is a reasonable starting point for research, given that human judgments are the logical gold standard for differentiation of cry and protophones, but the approach is scientifically incomplete in the absence of a systematic attempt to specify the acoustic bases upon which the judgments must be founded.

There are known difficulties in developing clear criteria for coding infant vocalizations (Kent and Murray, 1982; Lynch et al., 1995; Nathani and Oller, 2001). For example, Nathani and Oller (2001) addressed the fact that some fussy vocalizations (a category they deemed intermediate between cries and protophones) have substantial speech-like qualities (e.g., fussy canonical babbles), and as such they argued these utterances should be treated as protophones. Stark (1989) also argued for treating sounds as protophones to the extent that they had speech-like characteristics, even if they also had fussy or distress characteristics. Kent and Murray (1982) emphasized widespread disagreement among researchers on distinctions between speech-like and non-speech-like qualities of infant sounds. The point is highlighted in work by Green et al. (1998) who examined acoustic characteristics of sequences of cries, focusing on the first five cries versus the last five cries in a bout. The authors found significant changes in cry sounds across a bout, some seeming more cry-like than others. Porter et al. (1986) and Thoden and Koivisto (1980) also showed gradations in intensity of cry bouts based on presumed pain levels associated with invasive medical procedures.

### Definitions and Terminology for Distress and Non-distress Sounds in the Present Work: The Complexity of Infant Vocalizations and the Need to Focus on Prototypical Exemplars

Definitions are needed not only to differentiate cries from protophones and from intermediate fussy sounds, but also to provide labels that will help guide our stimulus selection. All the definitions we used are based on common English terms adapted for specific intentions in our research, and it should be emphasized that the terms are primarily of heuristic value.

We define the most salient and intensely distressful cry sounds as “wails” or “wail cries” (see Appendix 1: Acoustic exemplars, for a spectrographic display of item a; all the Appendices are in Supplementary Material, labeled as Supplementary Table 1; for a wave file of item a see Supplementary Data Sheet 1); each wail consists of a continuous phonation event perceived as intensely distressful. We use the term “whine” to designate a subcategory of fussy vocalizations, where there is also continuous phonation, but the auditory effect suggests lower intensity of distress than in wails (As for item a, see Appendix 1 in Supplementary Table 1 of Supplementary Material for the spectrographic display and see Supplementary Data Sheet 1 of Supplementary Material for the corresponding wave file). Many other fussy vocalizations include at least one glottal burst, a sharply produced egress, that sounds like a cough when isolated (Truby and Lind, 1965; Stark et al., 1975) followed by a short phonated nucleus; we term these fussy utterances “whimpers” (Appendix 1, item c), and mention them here for clarity of our approach, although they are not included among our stimuli in the present study, for reasons to be explained below. In accord with our definitions, wail cries can (though do not always) include glottal bursts and/or ingressive, spasmodic “catch breaths” (Truby and Lind, 1965; Stark et al., 1975; see Appendix 1, item d). The fact that whimpers and cries can include a wide variety of within-utterance combinations of these components (intense continuous phonation, glottal bursts, short phonated nuclei associated with glottal bursts, and catch breaths) creates considerable complexity and variability within different utterances interpreted as inherently distressful. In fact, there appear to exist a hundred or more different “formulas” for evolved distress sounds in the human infant (cry and whimper), involving variable sequences of these components (intense continuous phonation, glottal bursts, short phonated nuclei associated with glottal bursts, and catch breaths).

Our approach to this initial study takes account of the need to simplify the focus—with a hundred or more possible cry/whimper types, it is necessary to target a limited range of sounds. Nature fortunately provides a convenient prototypical target, the wail. Wails (consisting of a single phonated nucleus) are prototypical cries in that (1) a wail nucleus in *the only essential component for a sound to be judged as an intensely distressful infant vocalization*—glottal bursts, short phonated nuclei associated with glottal bursts, and catch breaths are unnecessary, and (2) *no vocalization can be judged as an intense cry in the absence of a wail nucleus* (whimpers lack a wail nucleus, which seems to account for their being deemed less distressful). In addition, (3) wails occur as common representatives of the cry class from the first day of life. It is also to our advantage to focus on wails in stimulus selection for highly distressful sounds, because the fact that wails consist of single phonatory events constrains the number of necessary acoustic analysis parameters, a number that can explode if utterances consisting of the many cry and whimper components (the hundred or more formulas) are brought into the picture.

Nonetheless within wails, there is still notable complexity. Even within a continuous phonatory event, major variation can occur in terms of shifts in vibratory regimes, from modal to loft to subharmonic to chaotic, etc. (Buder et al., 2008). Such shifts have been referred to in an early literature; Truby and Lind (1965), for example, categorized cry as including several phonatory types: normal (or modal) phonation, dysphonation (noisy or harsh), and hyperphonation (sounds at very high pitch). Early researchers (e.g., Wasz-Höckert et al., 1985) argued that these variations in cry were so substantial that research should be based on a consistent selection criterion to limit the variation, e.g., always selecting the first cry in a bout.

Furthermore, protophones are at least as complex as cries (Buder et al., 2013). They can include simple phonatory nuclei, or the nuclei can be combined with a wide variety of interruptions and/or modulations such as silences, friction noises, trills, bursts and so on. The formulas for protophones are many, and our experience suggests they outnumber the formulas for cry and whimper. Moreover, protophones can (like cries) include various phonatory patterns, including modal (normal), loft (falsetto), pulse (glottal fry), subharmonic, biphonation, and chaotic regimes (Buder et al., 2008). “Vocants” (or vowel-like sounds), the most prototypical of protophones in the first half year, are produced with modal phonation in the mid-range of f_0_ for each individual (Oller, 2000; see Appendix 1, item e). “Squeals” are produced at high f_0_, while “growls” are defined by very low f_0_ or by harsh voice quality. Periods of modal, loft, and fry can occur in a single infant utterance. In such cases, the most salient regime seems to guide coders’ choices in categorizing protophones as vocant, squeal, or growl.

In addition, movements of the supraglottal tract during phonation often interrupt phonation to create rhythmicity akin to syllabification, and such movements can occur in protophones even as early as 1 to 4 months (Oller, 2000). Such syllable-like modulations represent yet another way the formulas for protophones are complicated. By the second half year of life, both primitively articulated and well-formed canonical syllables can occur while producing protophones, whines, and even cries.

Consequently, as with cries and whimpers, it is important to focus on a simplified class of protophones for stimulus selection. Nature again provides a convenient and well-motivated choice for the prototypical protophone. Vocants are by far the most frequently occurring protophones (Nathani et al., 2006; Oller et al., 2013). They also consist most commonly of a single phonatory event that can be straightforwardly compared in acoustic terms with wails.

Finally, a subclass of vocants manifests negativity in its sound features—these vocalizations are whiny. Approximately 15% of continuously phonated protophones have this characteristic (Jhang et al., 2017). “Whines” can be thought of as sounds that would be wails if their distress expression were more intense. Whines are the prototypical sounds that occupy the middle of the continuum from wail to protophone.

We do not find a sharp categorical break along the vocal distress continuum—in fact different sounds grade from being perceived as highly distressful to not at all distressful, with no discernible discontinuity. Gradation of intensity in vocal signals is common across mammals (Marler, 1976; Jürgens, 1982; Ploog, 1992). Still, languages tend to provide labels for the ends of the continuum and for its middle, presumably because parents need convenient ways to refer to infant utterances.

In our work the terms wail, whine, and vocant are useful labels, but they are not necessary for the essential aspects of the work. We could just as well speak of intensely distressful sounds, moderately distressful sounds, and sounds expressing very low distress or none at all.

### Intuitive Identifiability of Cries and Protophones

Interestingly, despite the complexities of formulas for sounds on the continuum, human listeners intuitively perceive differences in infant vocalizations and show consistent judgments differentiating cries and protophones (Oller et al., 2013). With somewhat more explicit instructions than have been given in most prior research, Yoo et al. (2018) found very high agreement (*r* > 0.9) among five coders asked to count protophones and cries in 28 five-minute recording segments. Furthermore, the data showed that caregivers were more likely to (1) take vocal turns with the coded protophones and to (2) vocally overlap with the coded cries from the first months of life. Thus, both laboratory coders and caregivers consistently treated infant protophones and cries differently, a clear indication that infant signals contain reliable acoustic information indicating distress or lack thereof. Non-parent adult listeners were also able to identify cries versus whines selected from recordings of infants (Yoo and Bidelman, 2016). Research has also shown that parents are able to identify cries from their own infant among other cries of similar aged infants (Wiesenfeld et al., 1981).

As far as we know, there has been no attempt to directly account for the role that acoustic parameters play in the distinction between cries and protophones. Furthermore, since caregivers seem to intuitively judge varying *degrees* of distress in infant sounds in daily life, more systematic research is needed to investigate the link between perception of level of distress and acoustic correlates of perception along a continuum from cry to protophones. This line of work could lay important foundations for studies both on the development of speech infrastructure and for clinical studies focused on cry and speech-like vocalizations.

It is also of interest that perception even in infancy is acute. Many minimal speech sound contrasts have been proven discriminable by very young infants (Eimas et al., 1971; Eilers et al., 1981; Werker and Tees, 1984; Jusczyk, 1992). Furthermore, *in utero* experience has been reported to influence vowel perception in infants (Moon et al., 2013). Infants have even been shown capable of responding to sea gull calls without seemingly finding them aversive, perhaps because they resemble the infants’ own cries (Lange-Küttner, 2010). This acute discrimination and responsivity to a wide range of natural sounds suggests considerable human capacities for perception extending into many domains even in the first days of life. Perhaps then it should be no surprise that human adults are capable of acute perception of vocal distress.

Perception and production are distinct in many ways. One of these is that human infants are capable of discriminating among sounds they do not produce. Thus syllables discriminated by young infants in the above cited studies are not produced by infants until many months later if ever (see review in Oller, 2000). The present research on vocal distress perception is founded on the recognition that infant sound production is constrained to cries, protophones, and vegetative sounds in the first two months of life. This limited range provides the basis for useful signals to caregivers, who appear to have been evolved to recognize these sounds and discriminate among them as a basis for determining infant needs.

### The Dynamic Nature of the Perception of Infant Vocalizations and Evolutionary Requirements of Natural Signaling Systems

Our approach, being inspired in part by evo-devo tenets, is also intended to illuminate the potentially dynamic nature of perception and interpretation by caregivers. We assume that listeners who judge distress in infant vocalizations bring to bear a basic human capacity for recognition of distress, a capacity without which they would be unprepared for caregiving. At the same time there is reason to assume that the capacity for vocal distress recognition is not static, but rather that it is adjusted moment-by-moment in perceiving infant sounds and in interpreting them so that they can be used as a basis for adaptive caregiving. The adult perceptual system must, in accord with this reasoning, be active, and it must, whether tacitly or explicitly, be directed to *learning about the sounds of the individual infant* as that infant’s vocal tendencies mature.

We reason, then, that a normal adult perceiver, in the course of rating infant vocalizations with regard to distress, will tend to explore the acoustic parameters and adjust the basis for judgment during the task, rather than simply to apply a fixed set of acoustic criteria supplied innately. The notion of active perception was advocated by Gibson (1979), who argued perceivers are not passive, but instead adapt constantly to the needs of perception, turning their heads, focusing their eyes, moving in the direction of the signal, and so on. Even robotic systems can be shown to work best when they incorporate active perceptual features (Bajcsy, 1988).

Consequently, we included a large group of listeners to make judgments of vocal distress across a small set of carefully selected infant sounds in multiple trial blocks. This design allowed us to evaluate natural adjustments within individual listeners in how they made distress judgments across the task on multiple trial blocks with the same stimuli.

In addition, our strategy allowed us to assess differences across individuals in how they used the acoustic parameters to make their judgments. Natural selection of course requires variation on traits (Darwin, 1859), since any *uniform* trait has no basis for differentiation of fitness on that trait. The ability of human caregivers to judge functions of infant sounds is clearly a trait contributing to inclusive fitness of the parents themselves (Hamilton, 1963) because it provides a basis for their investment in their infants’ survival and reproductive success (Trivers, 1972). Our research will provide the first assessment to our knowledge of differences among human listeners in their judgments of the vocal distress continuum.

Similarly, our study will assess potential short-term adaptations of humans on the trait of vocal distress judgments by comparing judgments of listeners with and without experience in infant vocalization coding. Evo-devo tenets predict substantial adaptability on any trait in the context of experience that is relevant to application of the trait (Hall, 1992; Lickliter and Honeycutt, 2003; Müller and Newman, 2003; West-Eberhard, 2003).

### Summary of Rationale and Goals for the Present Study

Research has so far failed to establish acoustic criteria that define cry as distinct from protophones. Furthermore, no prior research has attempted to address the whole continuum of phonatory phenomena (from cry to protophones) that make it possible to reliably judge the level of distress in infant vocalizations. The goals of this study were to investigate the perceptual and acoustic properties of a carefully selected small set of prototypical infant protophones (specifically, vocants), cries (specifically, wails), and vocalizations of intermediate levels of distress (whines). We approached the study aware of the common shifts in vibratory regimes that occur within infant utterances, and we resolved to address vibratory regimes themselves as a key feature of our analysis. We sought to determine

(1) Reliability of vocal distress signaling in the first months as indicated by the extent to which a panel of adult listeners agree on level of distress for the selected stimuli, as manifested by inter-rater correlations, and the extent to which they show individual consistency as manifested by intra-rater correlations across the ten trial blocks;
(2) The acoustic parameters that best accounted for perception of level of distress, as indicated by multiple regression on listener ratings and acoustic parameter measures;
(3) The extent to which *individual* listeners maintained or changed their acoustic criteria for distress judgment across time, as indicated by intra-rater correlations with the acoustic parameter measures across multiple trial blocks;
(4) The extent to which *different* listeners used similar or different acoustic criteria to make their judgments of vocal distress, as indicated by comparisons of inter-rater correlations with the acoustic parameter measures; and
(5) The role of experience in infant vocalization coding on patterns of perception among the listeners.

## Materials and Methods

### Participants

#### Infants

We acquired stimuli from recordings of seven infants with normal hearing and no known developmental impairments. All parents completed a written informed consent for the recordings, approved by the Institutional Review Board of the University of Memphis. We included data only from newborns (0 to 1 months of age), consisting of protophones and distress vocalizations in this initial phase of our research on this topic for the following reasons: (1) The rate of occurrence of infant wail cries is at its highest in the newborn period, decreasing dramatically after 2 months (Wolff, 1969; Nathani et al., 2006), thus the newborn period is the easiest time to find samples of vocalizations across the cry to protophone continuum; (2) Significant neurological development occurs at around 2 months that may have impact on the form of infant vocalizations (Rochat, 1998); (3) Given that cries and protophones change under the influence of development and learning (Wilder and Baken, 1978; Stark, 1980; Koopmans-van Beinum and van der Stelt, 1986), newborn vocalizations may represent the most prototypical forms of distress signaling across the lifespan; and (4) In research on the origin of language, it is sensible to begin studies from as soon as infants can vocalize (Oller et al., 2016).

The stimuli from seven infants represents a limitation of the present study since there is no assurance that results based on this small set of infants can be generalized to the population at large. However, we noticed no obvious anomalies in any of the seven infants’ vocal patterns, as compared with the many other infants we have studied in numerous prior investigations. It was necessary to keep the number of infants small in the present study for practical purposes because we also sought utterances representing all three points on the distress continuum at two ages for each infant. More infants would have resulted in more stimuli for the listeners to judge, and it was desirable to keep the session lengths for listeners short.

#### Listeners

Participants were 39 adults (37 females and 2 males) with an average age of 27.4 years (*SD* = 5.1; range = 21 ∼ 38 years). All self-reported normal hearing and no history of neurological or cognitive deficits. Thirty-four participants were native monolingual speakers of American English. The remaining five spoke English and additional languages (e.g., Korean, Spanish, Hungarian, Hindi, Telugu, and Arabic). Thirty-six participants were graduate students at the University of Memphis and three were staff members in the Infant Vocalization Laboratory. Four participants (2 students and 2 staff) were parents. 49% (*n* = 19) of the listeners were graduate research assistants in the Infant Vocalizations Laboratory and thus had participated in systematic training in coding of infant vocalizations, including differentiating cry, whimper, and protophones, and had participated in prior coding studies. The remaining 20 had not had any training or experience in coding of infant vocalizations. The fact that almost all the listeners were female is another limitation of the study, although we presume an imbalance favoring females may be more advantageous than an opposite imbalance given the importance of maternal care in infancy.

All listeners completed a written informed consent for the experiment, approved by the Institutional Review Board at the University of Memphis.

### Distress Level Judgment Task and Acoustic Analysis Procedures

#### Recordings

All utterances were extracted from all-day LENA recordings from the University of Memphis archive, which made it possible to extract naturally occurring infant vocalizations from five-minute periods that had previously been coded by trained human listeners (Yoo et al., 2015). The LENA recorder is small enough to fit in a vest pocket of clothing for infants. The distance from the infant’s mouth to the microphone is ∼5–10 cm. The sampling rate is 16 kHz, providing adequate quality for human coding and acoustic analysis (for details on LENA recording, see Xu et al., 2014).

For a previous research project in our laboratory (Yoo and Oller, 2016), staff had previously selected 24 segments (each five minutes long) randomly from each LENA recording from seven infants, in addition to selecting the 10 segments that had yielded the highest infant vocalization rates according to the LENA automated analysis (Xu et al., 2014). Human-listeners had coded these 34 segments per recording, providing reliable indications for each five-minute segment regarding the number of infant vocalizations, both protophones and cries.

For the present study we selected utterances from these coded five-minute segments, locating them conveniently in the samples with the largest numbers of both cries and protophones as indicated by the coding. The number of cries in segments became the primary criterion, because cries were considerably less frequent than protophones throughout the data corpus, and often caregivers spoke over cries, making them unanalyzable by some of our acoustic methods. In general it was easy to find sufficient numbers of protophones in the same samples that had enough analyzable cries to meet our stimulus requirements.

#### Rationale for Focusing on a Restricted Set of Infant Sounds: Stimulus Selection

Since both protophones and cries are highly complex (see above), we limited the stimulus utterances to a small set of 42 exemplars (14 selected as wails, 14 as whines, and 14 as vocants) containing only phonation. No supraglottal articulations (Appendix 1, item f) or other phonatory interruptions were included. By selecting phonatory-only segments, we focused on exemplars that were particularly amenable to well-developed principles of acoustic analysis for all utterances across the entire continuum. Also the goal was to select the most prototypical types of vocalizations along the continuum.

The most prototypical protophones, are no-distress or very low-distress vocants. Vocants are the most prototypical protophones precisely because they are the most frequently occurring protophones, being far more frequent than squeals or growls, the other most prominent protophones of the first months (Oller et al., 2013). Vocants are differentiated from squeals and growls by consisting overwhelmingly of modal phonation (Buder et al., 2013; Appendix 1, item e), thought of as the default pattern of phonation, which provides another reason to think of vocants as prototypical, since speech consists overwhelmingly of normal phonation. Squeals diverge from the default pattern of phonation (modal), showing very high f_0_, often with loft. We excluded very high-pitched sounds from all three types of stimuli (wails, whines, and protophones). Growls were also excluded due to their characteristic pulse or rough (e.g., subharmonic) phonation.

Only utterances perceived as intensely distressful were selected as wails. In accord with our definitional criteria, the wail nucleus, the period of continuous phonation, contains the most prototypical distress indicators. Glottal bursts and catch breaths were excluded (Appendix 1, item a). Wails were selected as prototypical cries as indicated above because: (1) wail nuclei occur in all cries perceived as highly distressed, (2) no cry or whimper without a wail nucleus is perceived as highly distressed, and (3) wails occur frequently as cry sounds in early infancy.

Items selected as whines were interpreted intuitively as distressful, but less distressful than wails (Appendix 1, item b). The whines selected thus represented utterances presenting an intermediate level of distress between wails and protophones, and as with the other types, they included phonation only.

This selection method restricted the set of utterances to three prototypical vocal types—the most intense distress sounds in their simplest form (wail cries), the least distressful vocalizations in their simplest form (vocants), and the intermediate distress class of whines. The single dimension focused on here does not include the presumable opposite of distress, joy or positivity, because positivity is not reliably identifiable in very early infant vocalizations (Jhang and Oller, 2017).

We included infant utterances only when they were (1) highly audible and discernible and (2) produced without overlay by caregiver vocalizations or background noises. We excluded any utterance that (3) was perceived as so low in intensity that we deemed it would not tend to be noticed by caregivers, (4) was shorter than 400 ms or longer than 2000 ms, or (5) would have been deemed a squeal or growl.

From the seven infants at 0 and 1 months, the first author found a total of 422 utterances (∼10 utterances for each vocal type, infant, and age) meeting these criteria in the 14 recordings. In the first step after selecting the 422, utterances were proposed by the first author as pertaining to one of the three types. Then each utterance was rated by the first and last authors according to how well it represented the level of distress pertaining to the designated type using a 10-point Likert-type scale. For example, an utterance designated by the first author as a wail, would be ranked by both authors along a 10-point scale regarding how distressful it sounded, with 1 being most distressful (and thus most wail-like) and 10 being least distressful (and thus least wail-like); consequently the optimal wails were those that had rankings closest to one. For proposed vocants, the two authors ranked them in the same way, and the optimal vocants had the distress rankings closest to 10. For proposed whines, the two authors ranked them with the same scale, and the optimal whines had midpoint rankings, that is, 4 to 6. The final rankings for each utterance were based on a consensus of the two authors, who talked about their impressions together until reaching an agreed ranking for each utterance. The utterances best conforming to the expected perceived distress levels based on these rankings for each vocal type (wail, whine, vocant) for each infant at each age were those selected as final stimuli for the perception task and the acoustic evaluation (7 infants ^∗^ 3 types ^∗^ 2 ages = 42 utterances), with each infant contributing one wail, one whine, and one cry at each of the two ages. It should be added that the three labels are not important to our approach nor to our research questions—our interest here is in evaluating the continuum of vocal distress, not categories. Wave files for the 42 stimuli are found in Supplementary Material, Supplementary Data Sheet 2.

Of course it would have been possible to artificially adjust the stimuli or synthesize them so that certain acoustic parameters, for example Duration or Pitch were held constant. Such an approach may be useful in the future. However, we settled on natural stimuli for this initial study, since we wanted to assess all the primary acoustic parameters that might influence distress judgment, and recognized that the results of this initial work could provide a basis for selecting acoustic parameters for experimental manipulation in future work.

#### Listener Judgments for Distress Level

On each trial block (there were ten blocks for each participant, all 42 infant utterances occurring within each block), participants were asked to judge the level of distress for each of the 42 utterances presented in a different random order for each trial block. Listeners were *not* asked to categorize the sounds, and the terms cry, wail, whine, protophone, vocant, etc., were not mentioned in the instructions. A customized high-resolution slider scale was implemented in AACT (Action Analysis, Coding, and Training, Delgado, 2018). Wave files displayed in TF32 (Milenkovic, 2018), the acoustic analysis system invoked by AACT, were presented on a computer monitor, and the rating tool (from 0: no-distress to 100: very high distress) appeared on another. The task was to click with the mouse on the rating scale to designate a value from no distress to high distress. Participants read detailed instructions for the experiment, and the first author also verbally summarized the procedure before listeners began.

After a practice session with nine infant utterances that were not part of the test set and without feedback, each participant performed the actual judgments in 420 trials, where each stimulus utterance was judged 10 times (42 randomly ordered utterances × 10 blocked presentation trials). The ratings were obtained in a quiet room, and participants wore a headset to further minimize noise during the ratings. Participants were allowed to take breaks as needed. The task usually took about half an hour.

#### Confirmation That the Stimuli Represented a Broad Range of Distress

**FIGURE 1:**
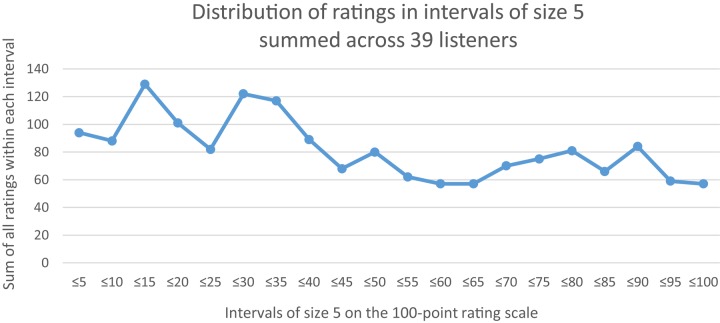
Number of mean ratings across the ten trials within all listeners across the entire 100-point scale in intervals of size 5. The figure illustrates that the entire rating scale was used by the listeners, that is, that ratings occurred within all the intervals of possible ratings. To understand the figure, note that each of 39 listeners produced 42 mean ratings over 10 trials on each of the 42 stimuli. Thus the figure represents 42 × 39 = 1638 mean ratings organized in 20 intervals. For example, the interval from 0 to 5 accounted for 94 mean ratings. The interval with the largest number of ratings (129) was 11 to 15, and the intervals with the smallest number of mean ratings (57) were tied at intervals 56 to 60, 61 to 65, and 96 to 100.

Our intention in stimulus selection was to represent a very broad range of vocal distress in order to evaluate listener agreement on distress judgments. Consequently, it is important methodologically that the ratings were in fact broadly distributed across the rating scale. Indeed, the stimuli represented an essentially continuous scale of vocal distress, as we had intended. To illustrate this methodologically important outcome, Figure 1 presents the distribution of the number of mean ratings *within* each of the 39 listeners summed within 20 intervals of size 5 across the entire scale. Table 3 in Results, provides the mean distress ratings for each of the 42 stimuli *across* all 39 listeners, again confirming that the entire scale was used.

### Acoustic Feature Determination

#### Hypothesized Predictive Acoustic Parameters

We began the acoustic analysis of the stimuli with 43 parameters representing a wide variety of possible predictors of perceived vocal distress, based on prior literature in acoustic evaluation of speech and especially infant vocalizations. These acoustic parameters are detailed in Appendix 2 in Supplementary Material with explanations of how we evaluated them in stages, reducing the number to the 9 that we deemed best representatives of parameters to predict the ratings of the listeners. Correlations among all 43 parameters and the distress ratings provided the first stage in selection, with high correlations being favored for selection. In the second stage, we considered pairs of parameters that showed both high correlations with the ratings and high correlations with each other, and we eliminated the member of each pair that showed the lower correlation with the ratings, especially in cases where there was strong reason to view the two parameters as conceptually similar, thus measuring the same sorts of properties. The 9 parameters remaining were: (1) Duration, (2) Average Pitch (f_0_), (3) Maximum Pitch (f_0_), (4) Maximum Amplitude (RMS), (5) Spectral Ratio, (6) Spectral Mean, (7) Spectral Dispersion (SD), (8) Periodicity, and (9) Number of Vibratory Regimes. The selected parameters were relatively independent of each other in that they were seen to measure conceptually independent properties (for example Duration was viewed as independent of Pitch and both were considered independent of Periodicity) and at the same time showed relatively high correlations with the ratings. See Appendix 2 for more details on the original 43 parameters and how we narrowed them down to 9.

#### Rationale for Vibratory Regime Analysis

Our approach included a strategy that we believe needs to be exploited in research on the vocal distress continuum and indeed in studies of infant vocalizations in general (Buder et al., 2008). The strategy focuses on the fact that vibratory regimes shift dramatically within infant utterances and on the apparent fact that the shifts themselves provide key information to the human perceiver. Here we provide rationale for this strategy.

Considerable research has been devoted to showing that the assumption of linearity of source and filter in vocalization (Fant, 1960; Stevens, 1998) is not generally valid, and particularly not with child vocalizations (Titze et al., 2008). According to Titze et al. (2008), source-filter interactions can produce violations of linearity. Interaction of glottal airflow with acoustic vocal tract pressures can result in non-linearities reflected in distorted harmonic frequencies. Non-linearity without source-filter interaction can be associated with subharmonics and biphonation.

A regime is a pattern of vocal fold vibration (Buder et al., 2008). There are three common registers for speaking: modal, pulse and loft (Hollien et al., 1977). Each register corresponds to a vibratory regime in the coding scheme to be utilized here, although we excluded utterances with loft from the stimulus set. In these regimes, vocal folds vibrate regularly and thus generate periodic waveforms. Bifurcations, i.e., sharp breaks from one regime to another, if they are not produced intentionally, are often considered pathological in adults (Herzel et al., 1994). While it was in fact common for early researchers to treat non-modal phonation types as indicative of neurological or structural pathology, recent studies have made clear that non-linear phenomena associated with several regimes occur regularly in vocalizations (cry and non-cry) of typically developing infants and children (Robb and Saxman, 1988; Mende et al., 1990; Buder et al., 2008; Fuamenya et al., 2015). Because these regimes substantially change harmonic patterns and energy distribution, we viewed it as necessary to account for regimes in seeking acoustic features that signal infant vocal distress.

#### Regime Segmentation of Each Utterance

Segmentation was performed within each utterance to designate the vibratory regimes listed below. Narrow (10–30 Hz bandwidth) and wide band spectrographic (300–500 Hz bandwidth) displays were used to determine variations in regimes within each selected utterance. We used both visual (i.e., spectrographic) and auditory stimulus information to identify regimes. For example, if subharmonics appeared in a very short (<50 ms) segment of a spectrogram, but we did not hear the distinctive period doubling (a specifically rough quality) that typically accompanies subharmonics, we did not label that brief segment as subharmonic.

For the purposes of the present study, we began with a regime scheme utilized previously in our laboratory, but simplified it after initial analysis revealed only a few categories had driven the rater judgments. We settled on four regimes (#1 – 4; for details, see Buder et al., 2008), and two types of modulations occurring during regimes (#5 and 6; for details, see Buder and Strand, 2003).

1. Modal: The modal regime is the typical phonatory pattern of speech, showing regular vocal fold vibration, with harmonics at regular multiples of the f_0_.
2. Aperiodic: This regime involves non-harmonic or harmonically unclear periods (i.e., chaos) or non-periodic extra harmonics (i.e., biphonation).
3. Subharmonic: This regime is defined “by the abrupt appearance in the narrow band spectrogram of intervening harmonic doubling, tripling, or even higher integer multiples in relation to the surrounding set” (Buder et al., 2008, p. 7).
4. Pulse: The pulse regime is associated with low f_0_ and often low intensity. Pulse is defined “by the appearance of very closely spaced harmonics often resulting in temporal resolution of individual glottal pulses in the waveform and sometimes also the spectrogram, and a clear perception of a low “zipper-like” quality (Buder et al., 2008, p. 6).
5. Trilling: This modulation does not refer to tongue or lip trilling, but to an effect generated at or near the glottis at modulation frequencies similar to those of tongue or lip trills.
6. Flutter: This code indicates modulations in f_0_, amplitude, or both, occurring at rates faster than syllables but slower than jitter/shimmer. Buder and Strand (2003) delineate three different types of modulations (tremor, flutter, and wow).

The inclusion of vibratory regime categorization within each of the utterances formed the basis for important new insights (as we hypothesized it might) about how vocal distress is judged by human listeners. In particular the Number of Regimes itself was selected as one of the 9 most predictive parameters. Four of the other selected parameters [Spectral Ratio, Spectral Mean, and Spectral Dispersion (SD) as well as Periodicity] were all measured regime specifically, that is events occurring within regimes (not across entire utterances) proved to be far more predictive than characteristics of whole utterances.

### Statistical Analysis

In accord with the 5 research questions listed above under Summary of Rationale and Goals for the Present Study, we conducted statistical analyses for each of the following questions:

1. On reliability of vocal distress signaling: The extent to which listeners agreed with each other (*inter-rater agreement*) on the ratings of vocal distress was assessed by comparing correlations between mean ratings for the 42 stimuli across the listeners. Similarly the extent to which individual listeners were consistent across trial blocks in levels of ratings for the stimuli was tested by comparing correlations of ratings across trial blocks (*intra-rater agreement on the ratings*).
2. On acoustic parameters that best accounted for perception: Multiple linear regression was used to determine the most predictive acoustic features for distress-level judgments and to provide perspective on possible unique strategies of listeners in judging distress level of vocalizations based on acoustic factors.
3. On the extent to which *individual* listeners maintained or changed their acoustic criteria: The extent to which listeners were consistent in their *own* ratings across 10 trial blocks for the 42 utterances (*intra-rater agreement on use of the acoustic parameters in rating*) was assessed by comparing correlations across the 10 trial blocks for each rater. To determine whether listeners varied in which acoustic parameters they used to judge vocal distress across trial repetitions, we computed correlations between each listener’s distress ratings and each acoustic parameter and then used a Cox and Stuart (CS) test for trend (Cox and Stuart, 1955) (see Supplementary Material, Appendix 3 for details).
4. On the extent to which *different* listeners used similar or different acoustic criteria: To evaluate *inter-rater differences on use of the acoustic parameters in rating*, we conducted an evaluation based on a permutation-type test of correlations between each listener’s ratings and each acoustic parameter. The procedure involved randomly varied resampling with replacement (a bootstrapping method) to allow comparisons of a large number of pairings of subgroupings of the correlations of the 39 listeners. More details on the permutation procedure and associated tests to assess possible inter-rater differences can be found in Appendix 3.
5. On the role of experience: To test whether experienced and inexperienced listeners differed with respect to how they relied on the acoustic parameters in making judgments of vocal distress, we computed mean correlations for each of the listeners and compared the correlations of the experienced and inexperienced listeners using a Wilcoxon test on each of the acoustic parameters. This is a non-parametric test that is preferable in this case to *t*-tests, given violations of the distributional assumptions of the latter. Family wise multiple comparisons (*p* ≤ 0.05; Bonferroni correction) were made in R on distress-level judgments to assess possible differences between the inexperienced and experienced listeners on ratings of levels of distress in the infant sounds.

## Results

### Consistent Ratings for Perception of Level of Distress

We began by determining the mean ratings for the 42 stimuli across all 10 trials for each rater—these are the mean *individual* ratings. We then evaluated how well different listeners agreed with each other in judging level of distress on the 42 infant utterances, finding that the mean Pearson correlation between all possible pairings of the 39 mean *individual* ratings (*n* = 38+37+36…) was very high, 0.92 (range 0.78 – 0.98). Even the lowest of these inter-rater correlations was significant at *p* < 0.00001. Another measure of inter-rater agreement was the correlation between the mean *individual* ratings of each one of the listeners and the mean for all the other listeners (38 pairings for each of the 39 listeners): this mean correlation across the 39 listeners was 0.96, and even the lowest (range 0.91 – 0.99) corresponded to *p* < 0.00001.

Individual listeners also showed high consistency across stimulus repetitions, with mean intra-rater agreement for all possible pairings of the 10 trial blocks (*n* = 9+8+7…) at 0.85 (range 0.57 – 0.94 across the 39 listeners), and again the lowest correlation was highly statistically significant (*p* < 0.0002).

The numbers of parents (4) and males (2) were too small for conclusive comparisons, but the results for the parent and male raters were quite similar to those of the other raters, and a specific analysis revealed no statistically significant differences from the larger group of non-parents and/or females.

A secondary point about agreement in these data concerns how the individual listeners used the 100-point distress scale and the extent to which they differed in rating utterances at high or low levels on the scale. The mean rating for the 39 listeners across the 42 utterances was 45.8 (*SD* = 7.2, range = 31.1 – 64.9, coefficient of variation 7.2/45.8 = 0.16). If we take the mean intra-rater coefficient of variation (CV) across the 10 trial blocks (0.09) as an indicator of rating noise, there remained discernible bias across listeners exceeding the rating noise, because the inter-rater CV at 0.16 was 0.07 (∼ 78%) higher than the intra-rater CV.

### Acoustic Parameters Predicting Level of Distress

**FIGURE 2:**
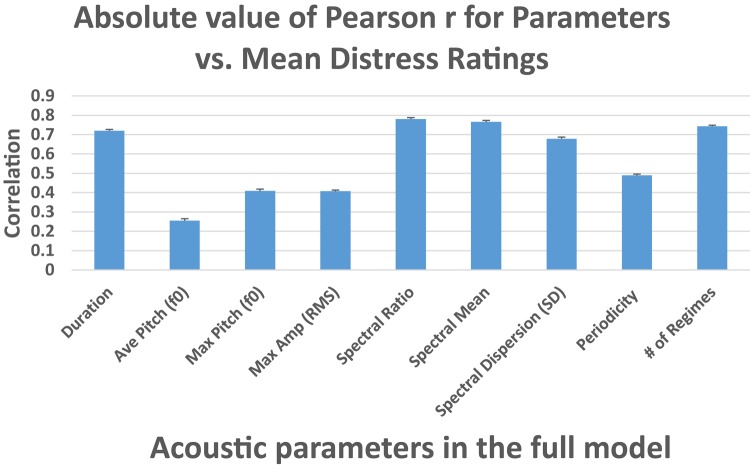
Pearson correlations between each of the nine acoustic parameters selected for the full model and the mean perception ratings of distress level. Average Pitch (f_0_ mean) represents mean fundamental frequency within each utterance. Max pitch represents the maximum f_0_ within each utterance. Max amplitude (peak of the root-mean-square amplitude) represents maximum amplitude (in volts) across each utterance. Spectral ratio represents the ratio for each utterance of spectral energy below 2 kHz to the energy above 2 kHz in the regime segment with the minimum ratio. Spectral mean represents the maximum mean of spectral concentration (long-term spectral average) in kHz across the regime segments in each utterance. Spectral dispersion (SD) represents the maximum standard deviation of spectral concentration in kHz across the regime segments in each utterance. Periodicity represents the minimum cepstral peak prominence in dB across the regime segments in each utterance, a measure of periodicity. Number of regimes represents the number of regime segments within each utterance. Error bars = ± 1 SEM.

Nine acoustic parameters were selected from among the original set of 43 as best possible predictors of the listeners’ ratings (see section “Materials and Methods” and Appendix 2). Pearson correlations between these nine acoustic measures and the mean distress ratings are displayed in Figure 2.

A full model multiple regression analysis of all nine parameters indicated that only Duration and Number of Regimes were significant predictors of the perceptual judgments (see Appendix 5 for an analysis of the relation between Duration and Number of Regimes). Diagnostics indicated collinearity in the full model, encouraging us to consider systematic ways to optimize the analysis by further reducing the number of predictors.

The adjusted R^2^ for the full model multiple regression was 0.80. To assess the relative importance of each predictor, we computed standardized coefficients (Table 1), which represent the mean change in the response given a one standard deviation change in the predictor. The original scales of the predictors are not represented in these estimates, so the effect of each predictor can be compared directly. The absolute values of the four highest standardized coefficients indicate the largest effect sizes of the acoustic parameters in predicting distress ratings: Number of Regimes, Duration, Spectral Ratio, and Average Pitch (f_0_). These four parameters and their coefficients are displayed in Table 2.

**Table 1:** Standardized coefficients and relative contribution to the full model of each acoustic predictor of distress ratings (^∗^*p* < 0.05, ^∗∗^*p* < 0.001).

Predictors	Standardized (β) coefficient	*p*-value
Intercept	47.0	<0.00001
Duration (ms)	8.0	**0.003**^∗∗^
Average pitch (f_0_)	6.2	0.13
Max pitch (f_0_)	−1.8	0.67
Max amplitude (RMS)	2.4	0.28
Spectral ratio	−6.9	0.10
Spectral mean	2.3	0.65
Spectral dispersion (SD)	2.4	0.59
Periodicity	1.6	0.64
Number of regimes	9.1	**0.03**^∗^

Statistics for the parameters of the best model predicting the ratings by the acoustic parameters in terms of lowest AIC (Akaike information criterion, which estimates relative quality of alternative models) are shown in Table 2. The R^2^ for the best model was 0.84. A backward selection method was used to determine the best model. In this procedure, a linear model was iteratively fit, and predictors were omitted from the model on each step based on lowest AIC. Backward selection also helped eliminate highly intercorrelated predictors of the full model. Max Pitch (f_0_) and Average Pitch (f_0_) were correlated at >0.8, and thus it may be justified to have Average Pitch (f_0_) represent a general f_0_ parameter. Similarly the three spectral parameters [Spectral Ratio, Spectral Mean and Spectral Dispersion (SD)] were inter-correlated at >0.8, and thus it seems reasonable for Spectral Ratio to be treated as representing a general spectral concentration measure. The values for these primary predictive parameters on each of the 42 stimuli are provided in Table 3.

**Table 2:** Parameters selected in the backward selection method model for predicting rated level of distress based on acoustic parameters.

	Mean (SD)	β	*p*-value
Intercept	NA	−19.0	0.22
Duration (ms)	1030.6 (450.9)	0.02	<0.0001
Average pitch (f_0_)	479.8 (66)	0.09	<0.025
Spectral ratio	1.77 (1.3)	−0.92	<0.001
Number of regimes	2.4 (1.4)	7.0	<0.003

**Table 3:** Mean distress ratings of the 39 listeners on the 42 stimuli.

No.	Mean distress ratings (0 to 100)	Duration (ms)	Average pitch (Hz)	Spectral ratio	Number of regimes
1	3.93	759	318	19.8	1
2	11.30	591	397	21.2	1
3	11.30	701	332.3	34.8	1
4	13.83	679	357.4	23	1
5	14.19	706	396.4	19.7	1
6	14.62	570	408.9	26.2	1
7	15.05	536	416.1	14.2	1
8	16.23	547	430.1	6.3	2
9	16.79	452	420.6	8.8	1
10	20.86	762	372.8	2.3	1
11	22.15	544	363.9	12.2	2
12	26.11	73	298.2	10.7	2
13	26.79	996	396.3	13.8	1
14	27.10	860	459.5	21.7	1
15	31.03	633	437.6	5	4
16	31.37	740	436.5	16	3
17	33.89	781	372.6	10.5	2
18	36.48	615	382.7	1	2
19	36.71	650	474.5	12.3	1
20	38.99	1079	366.5	6.6	2
21	39.81	1085	479.8	18.4	1
22	40.12	1964	494.6	23.7	1
23	40.77	836	396.4	6.7	1
24	41.39	1512	500.8	12.9	1
25	44.85	1096	316.3	4	2
26	46.84	707	509.2	12	3
27	51.89	855	385.4	−1.7	1
28	58.34	1401	390.9	−3.1	3
29	68.08	1976	428.5	−2	1
30	70.84	853	442.7	0.2	3
31	71.22	1252	435.9	9.9	3
32	76.26	1206	432.3	−12.3	3
33	77.97	1281	383.8	2	3
34	78.77	815	451.4	−1.4	3
35	79.42	1215	386.1	−8.1	3
36	79.51	1712	441.8	−9.6	3
37	79.93	1597	424.2	0.2	3
38	80.31	988	505.5	9.5	3
39	83.44	1743	373.7	−4.9	3
40	83.97	2000	384.9	−15.1	5
41	90.70	1361	378.3	−3.1	4
42	91.29	1891	423.6	−8.9	5

It is notable that in the backward selection outcome (Table 2), all four of the final predictors were statistically significant, and they showed the four highest standardized coefficients (i.e., the highest effect sizes) in the full model (Table 1).

### Possible Differences Within Listeners (an Intra-Rater Evaluation) in How the Acoustic Parameters Were Used to Rate Infant Distress

To determine whether listeners varied in the extent to which they relied on the acoustic parameters across the 10 trial blocks, correlations for the ratings of each listener on each trial block with each acoustic parameter were computed, and a Cox and Stuart test for trend was conducted (see section “Materials and Methods” and Appendix 3 for details). 12 of the listeners were consistent across the 10 trial blocks for the nine acoustic parameters, showing no case where they significantly varied across trial blocks on any parameter. However, 27 listeners varied significantly in how they made their judgments based on the acoustic parameters across the 10 trial blocks (i.e., for each of these listeners, at least one parameter showed a monotonic trend in one direction or the other). Number of Regimes was the parameter on which listeners changed most in the way they made their judgments across the trial blocks, with 10 out of 39 listeners showing statistically significant trends. A chi-square test showed a significant difference from chance (*p* = 0.011) for Number of Regimes across listeners (Table 4, column 3). Other parameters with *p* < 0.05 for the 39 listeners were Max Amplitude (RMS), Spectral Dispersion (SD), and Periodicity.

**Table 4:** Intra-rater differences across 10 trials.

Acoustic parameter	Number of listeners out of 39 with significant trends of variation across 10 trials	Chi-square test for intra-rater variation across 10 trials	
		Chi-square (*p*-value)	Effect size (w)
Duration	5	1.47 (0.23)	0.19
Average pitch (f_0_)	7	3.22 (0.07)	0.29
Max pitch (f_0_)	7	3.22 (0.07)	0.29
Max amplitude (RMS)	8	4.22 (0.04)	0.33
Spectral ratio	6	2.30 (0.13)	0.24
Spectral mean	6	2.30 (0.13)	0.24
Spectral dispersion (SD)	4	4.11 (0.04)	0.32
Periodicity	9	5.28 (0.02)	0.37
Number of regimes	10	6.40 (0.01)	0.41

*Column 2: Number of listeners out of 39 who showed a significant trend by Cox and Stuart tests, changing correlations between distress ratings and acoustic parameters across 10 trial blocks. Column 3: p-values from chi-square tests indicating significance levels for intra-rater variation across 10 trial blocks in the degree to which their distress ratings correlated with each acoustic parameter. df = 1, N = 39. Column 4: effect sizes (w) from chi-square tests indicating the magnitude of effects.*

### Possible Differences Across Listeners (an Inter-Rater Evaluation) in How the Acoustic Parameters Were Used to Rate Infant Distress

Table 5 shows the extent to which listeners varied with respect to each other in how they relied on the acoustic parameters to make their judgments of vocal distress, based on the permutation test described in Methods and in detail in Appendix 3. So, for example, as indicated in column 2 of Table 5, the acoustic parameter Duration yielded ∼2% cases out of 9752 permutations (all the tests were targeted for n ∼10,000 permutation, and all included >9700 trials) where the null hypothesis (that the randomly selected groups did not differ in their correlations with the acoustic parameters) was rejected at α = 0.05, whereas for the parameter Spectral Ratio, there were ∼20% where the null hypothesis was rejected out of the 9761 permuted comparisons. These data indicate very strong differences across listeners on usage of some of the parameters, namely highly significant differences in the correlations between different listeners’ judgments and the parameters Max Pitch (f_0_), Spectral Ratio, Spectral Mean, Spectral Dispersion (SD), Periodicity and Number of Regimes. In five of the six cases of significantly different usage of the parameters by the listeners, chi-square tests showed *p* < 0.00001, indicating inter-rater variation on use of the acoustic parameters was highly significant. Also the three other parameters [Duration, Average Pitch (f_0_), and Max Amplitude (RMS)] differed significantly from those listed above in that they showed significantly *fewer* differences from chance, indicating a lesser tendency for inter-rater variation in how the acoustic parameters were used in ratings.

**Table 5:** Inter-rater differences across 39 listeners.

Acoustic parameter	Proportion of trials failing to reject the null hypothesis in the permutation test	Chi-square test for inter-rater variation		
		chi-square (*p*-value)	effect size (w)	*N*
Duration	0.98	117.54^^^	0.11	9752
Average pitch (f_0_)	0.98	89.33^^^	0.10	9757
Max pitch (f_0_)	0.92	79.01^a^	0.09	9747
Max amplitude (RMS)	0.97	74.22^^^	0.09	9778
Spectral ratio	0.80	997.68^a^	0.32	9761
Spectral mean	0.94	9.14 (0.003)	0.03	9756
Spectral dispersion (SD)	0.77	1345.15^a^	0.37	9770
Periodicity	0.92	57.50^a^	0.08	9747
Number of regimes	0.90	201.39^a^	0.14	9734

*Column 2: Proportion of cases (out of ∼10,000) for the permutation test failing to show <0.05 level differences in correlations between distress ratings for the infant utterances and the acoustic parameters across randomly selected rater groupings. Column 3: chi-square and p-values from chi-square tests indicating the probability that each acoustic parameter was used the same way across listeners. ^a^indicates that the proportion was significantly lower than expected by chance at p < 0.00001 (df = 1), that is, that listeners tended significantly to differ from each other on how they used the acoustic parameter in judging distress. ^^^indicates that the proportion was significantly higher at p < 0.00001 (df = 1) than the proportion for any of the cases that were significantly lower than expected by chance, the ones with superscript a. Thus 6 of the 9 parameters showed significantly more inter-rater differences than expected by chance, and the remaining 3 showed significantly fewer inter-rater differences than the other 6. Column 4: effect sizes (w) of the tests. Column 5: Numbers of cases of permutation tests.*

### The Role of Experience in Coding on Distress Ratings

We compared the ratings of the 19 listeners who had experienced some infant vocalization training and had coded prior samples (identifying vocal types) with those of the 20 listeners who were inexperienced in infant vocalization research. By a family wise (Bonferroni corrected) comparison, there was no difference between ratings of the experienced and inexperienced listeners.

**FIGURE 3:**
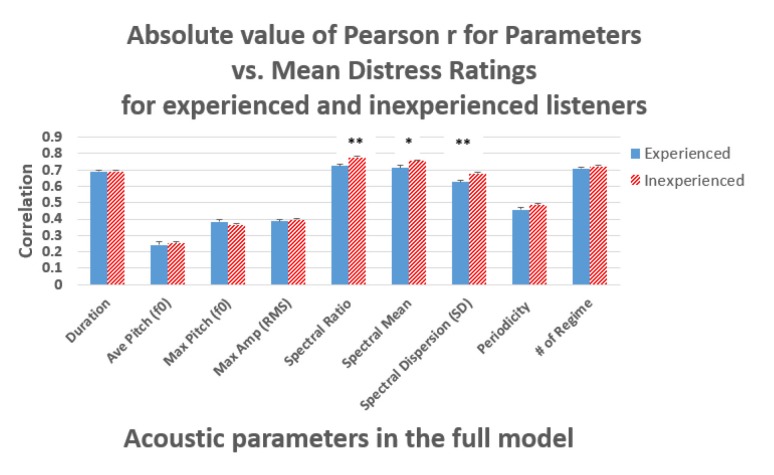
Pearson correlations between each of nine acoustic parameters and the mean ratings of distress level by experienced and inexperienced listeners. Spectral Ratio, Spectral Mean, and Spectral Dispersion (SD) were significantly different across groups (see Appendix 4 to view statistics on all 9 parameters). Error bars = ± 1 SEM. ^∗^*p* < 0.05, ^∗∗^*p* < 0.001.

For each acoustic parameter, we computed correlations with the distress ratings of the listeners within each group (see Figure 3). To determine whether the mean correlations with the acoustic parameters differed, we conducted a Wilcoxon test (further details in Appendix 4, Supplementary Material). For example, the *p*-value for the difference in correlations between ratings and Duration, for experienced listeners versus inexperienced listeners was 0.79, indicating no evidence that experience predicted listeners’ use of this acoustic parameter. However, all three spectral concentration parameters [Spectral Ratio, Spectral Mean, and Spectral Dispersion (SD)] showed reliable differences across groups.

## Discussion

### Summary of Results

(1) Listener agreement was high on degree of vocal distress, with mean r > 0.9 for both inter-rater and intra-rater evaluations; (2) statistically significant acoustic predictors of vocal distress were number of vibratory regimes within utterances, utterance duration, spectral ratio (spectral concentration) in vibratory regimes within utterances, and mean pitch; (3) >two-thirds of the individual listeners significantly modified their acoustic criteria for distress judgments across the ten trial blocks, suggesting an interaction between infant vocal distress expression and active perception by potential caregivers, who appear to act as learners continuously seeking to interpret infant signals, (4) while showing overall similarities in judgment criteria, different listeners also showed significant differences on 5 specific acoustic criteria used in judgments of vocal distress, and (5) listeners who were both experienced and inexperienced in infant vocalizations coding showed high agreement in rating level of distress, but differed in the extent to which they relied on three of the acoustic parameters in making the ratings.

### Inter- and Intra-Rater Agreement in Rating Vocal Distress and the Origin of Language

Our study offers an expanded view of how vocal distress is expressed in human infancy and how well it can be recognized by (primarily female) adult listeners. However, our intentions are driven by interests in the origin of language (for expanded perspectives on this point see Appendix 6, Supplementary Material), and consequently we have addressed infant vocalizations that both do and do not express distress. Protophones, in particular, are sounds that infants can produce with or without signs of distress. The protophones have been argued to manifest a capacity for voluntary vocalization that lays a foundation for language (Oller et al., 2013). In studying vocal distress, we address a continuum from sounds that show maximum distress (cries) to sounds that show minimum distress (vocants, the most common type of protophone). We also included in our study sounds that are intermediate in distress, referring to these as whines. No prior published study has ever addressed the whole continuum of infant vocal distress sounds to assess perceptual consistency and acoustic predictors. It may be useful to point out that the strict limitation of cry/wail to expression of distress is limited to early infancy, since adults clearly have much more flexible control of crying which is sometimes produced in circumstances of relief or joy (for perspectives on this point both in humans and other species, see Appendix 7, Supplementary Material).

A key finding of the present study was that human listeners, whether experienced in research on infant vocalizations or not, showed remarkable agreement in judging degree of distress across the continuum of infant vocalizations. These results suggest that the infants’ signaling of distress is very reliable. It appears that humanity has been evolved to have strong intuitive awareness of vocal distress in infants, a capability that would seem to be critical in intuitive parenting (Papoušek and Papoušek, 1987).

Notably, we also found that listeners’ perceptual judgments of vocalizations showed short-term changes (within the ∼30 min of task time) in the degree to which they relied on the acoustic parameters in rating infant distress. This finding suggests that human listeners, even without feedback, engage in judgments of distress variably, as if exploring possible ways of making distress judgments moment by moment and thus of probing to understand infant needs or seeking to discern infant fitness. The apparent exploratory tendency is consistent with expectations based on theories invoking the idea of active perception (Gibson, 1979; Bajcsy, 1988).

Furthermore, while listeners showed strong agreement with each other in rating infant distress, they differed in how they used acoustic cues to achieve those judgments. Of particular interest, we found that experienced and inexperienced listeners differed in how they utilized three of the acoustic parameters. This latter finding was unexpected, because the training and coding of the experienced listeners never explicitly focused on the acoustic parameters that differentiated the listener groups.

### Most Potent Acoustic Predictors

Our research also sought to determine the most potent acoustic predictors of infant vocal distress in prototypical cries (wails), whines, and protophones (vocants). In our initial acoustic explorations, we had speculated that wailing (i.e., crying without glottal bursts or catch breaths) is signaled primarily by spectral ratio (also called spectral concentration), such that energy levels (reflected in amplitudes of the spectrum) are relatively high above 2 kHz in cry and relatively low above 2 kHz in protophones. Gustafson and Green (1989) previously suggested that more energy at higher frequencies contributed to judgments of greater aversiveness among cries, but protophones were not evaluated. Notably this spectral concentration feature of cries that we explored often occurred *within a single vibratory regime segment* of an utterance, and usually did not characterize the utterance as a whole. Our speculation inspired us to consider parameters reflecting spectral concentration *within particular regime segments* as providing potentially important predictors of vocal distress. Indeed, the low/high spectral ratio (the Spectral Ratio variable in the present work) in the regime segment with the lowest ratio turned out to be one of the strongest predictors of the ratings of vocal distress.

Not only the low/high spectral ratio, but also the other two spectral parameters [the maximum mean and maximum standard deviation of the regime-specific long-term spectral average, our Spectral Mean and Spectral Dispersion (SD)] were strongly (∼0.7) correlated with rater judgments of distress. Moreover, the three spectral parameters were precisely the three that were utilized significantly differently in making distress judgments by listeners who had experienced infant vocalizations training and those who had not. Specifically, the inexperienced listeners appeared to rely *more* on the spectral parameters than the experienced listeners. Furthermore, all three spectral parameters showed significant variation *across* listeners in the inter-rater agreement data. We are inclined to speculate that spectral parameters constitute a factor that tends to attract strong attention in some listeners, especially in listeners who have not previously engaged in formal coding of infant vocalizations. Such strong attention in some, but not all listeners, may yield variable responses across listeners. Perhaps training and exercise of coding in infant vocalization tends to balance the attention of listeners slightly away from spectral parameters and toward factors that are more stable in indicating distress [Duration, Average Pitch (f_0_), and Maximum Amplitude (RMS) of utterances]; this was reflected by little or no tendency for differences among listeners regarding the influence of these factors on ratings. Indeed these three factors showed the lowest tendency among the nine acoustic parameters in the full model to differ in their impact on judgments of different listeners (Table 5). Our speculations about the role of spectral parameters in distress judgments and especially its role in differences among experienced and inexperienced listeners clearly call for additional research for validation.

Several other factors were also highly predictive of listeners’ percepts, most notably, Duration. Referring to the original labels used in stimulus selection, wails were longer than whines, which were longer than vocants. Importantly, Duration might have proven to be an even more salient predictor of distress judgments had we not limited our stimuli to 400 to 2000 ms. Lest one think, however, that duration always determines the distinctions, there were two wails in the sample that were shorter than two of the vocants, and yet listeners unambiguously rated distress levels of the short wails in the range of the other wails and the long vocants in the range of the other vocants (*p* < 0.001).

Another factor that contributed clearly to the prediction of vocal distress was Average Pitch (f_0_), which appears to correspond with a prior finding that cries with higher pitch are judged to be more aversive (e.g., Zeskind and Marshall, 1988). This factor correlated at >0.8 with Max Pitch (f_0_), and consequently we interpret the prediction of distress ratings as being related to f_0_ in general. Ours is, however, the first direct comparison of acoustic features in cries and protophones and thus suggests, for the first time directly, that f_0_ predicts vocal distress across the whole continuum of infant vocalizations. However, the present study only responds to part of the relevant question, because we again artificially restricted the f_0_ range by not including squeals among the protophones nor hyperphonation among the wails. In a subsequent study we plan to address the roles of loft and of pulse, as well as other rough phonatory features, in the perception of vocal distress in infancy.

The Number of Regimes (i.e., number of vibratory regime tokens) within utterances also contributed significantly to rating degree of distress. Gustafson and Green (1989) showed that adult listeners perceived infant cry as more aversive as a function of the amount of dysphonation in the rated utterances. We specifically took account of dysphonation within each utterance by coding segments as aperiodic or subharmonic in our regime coding scheme. The Number of Regimes, therefore, often reflected the presence of dysphonation.

Our opinion emphasizing the complexity of vocal distress expression in human infants has been amplified by the experience of studying these utterances (and hundreds of others) individually. The current acoustic analysis, which included segmentation of each utterance into vibratory regimes, suggested that several parameters are involved in judgments of distress, even in this restricted set of phonatory-only vocalizations. The complexity of the determining parameters may be even greater than we have been able to show with the analysis presented here. For example, for every utterance preselected as a wail, there was at least one regime segment >200 ms (and often several) for which the judgment “wail” did not apply according to the two stimulus selectors (first and last authors) when the segments were played back in isolation. Instead, these judges deemed these segments to be in modal voice, thus corresponding to vocant-like phonation. Often there were multiple such segments within a wail, and sometimes they were >500 ms. Also, in all but one of our wail utterances, there was a notable regime segment marked by the acoustic analyst either as aperiodic or as including subharmonics, designations that presumably would have been called dysphonation in most of the earlier literature. These regime segments did not, however, by themselves necessarily determine a judgment of wail—i.e., if the regime segment was played back in isolation, it often was not judged by either of the two stimulus selectors as unambiguous wail—rather these segments sometimes sounded strained or growly, but not unambiguously cry-like. The most common pattern of wail included both beginnings and endings of >100 ms that were judged unambiguously in isolation to consist of modal voice, i.e., they sounded like vocants. During the intervening regime segments, there was typically at least one dysphonated segment, that did not necessarily sound like wail in isolation, but in combination with the adjacent segments, was judged as unambiguous wail. Consequently, we infer that the great majority of our wail utterances were characterized by a strong contrast between at least one regime segment of dysphonation and surrounding segments of modal phonation (vocant).

An interesting possibility involving another change across time within distress utterances is suggested by work of Wermke et al. (2002), who described cry as often including a rise-fall contour. The first and last authors of the present study evaluated the utterances preselected as wails regarding this factor and found that only half of the 14 wails showed a rise-fall pattern. The remainder showed flat, complex, or rise-then-flat patterns. Five of the 14 utterances that had been preselected as vocants also showed a rise-fall pattern, with the remainder showing flat, complex, or rise-then-flat patterns. Thus, the hypothesis that a rise-fall pattern would be a strong predictor of wail was not straightforwardly supported in this small sample of utterances. This impression is fortified by Várallyay and Benyó (2007), whose data suggested that only about a third of cry utterances have the rise-fall contour. However, the possibility remains that melody contour may play a significant role in distress perception. A much larger study will be needed to evaluate this possibility. At present, it would appear that overall contours are much less influential in determining judgments of distress than the factors revealed by our analysis.

### Importance of the Vibratory Regime Analysis

We hasten to emphasize that much of the pattern of results depends upon the vibratory regime analysis. Prior research has not taken this approach in comparing cries and protophones. While prior research has *taken notice of* regimes (e.g., Fuamenya et al., 2015), it has not taken systematic account of them in assessing predictive power of factors such as aversiveness of or distress manifest in cries *and* protophones. Our present data suggest that future research on acoustic markers of distress should take account of vibratory regimes. Without regime analysis in the present work, we might have concluded that Duration and Pitch (f_0_) were the most important factors in the judgment of distress. No indications of differences across listeners, across trials, or across experience levels would have been revealed. In fact, Duration was the only significant predictive factor in the initial regression analysis other than Number of Regimes. As such, had we not considered regime-segment-specific factors, Duration might have appeared to be the only important predictor of distress ratings (see Appendix 5, Supplementary Material).

However, after including the regime-segment-specific factors, much more varied and interesting influences were revealed: (1) Regime-specific Spectral Ratio proved to be an especially strong predictor; (2) Number of Regimes itself was revealed as a significant factor along with Pitch (f_0_); (3) Listeners differed across ten 10 trial blocks (within only about half an hour) on how highly their judgments correlated with particular acoustic parameters; (4) Listeners proved to differ in their degree of correlation between ratings and the acoustic parameters; and (5) Experience proved to have a notable effect on how rating judgments were made. All these patterns would have been gone undetected if the analysis had ignored vibratory regimes.

## Ethics Statement

All the work reported here was approved by the University of Memphis Institutional Review Board for the Protection of Human Subjects.

## Author Contributions

HY designed the study, coordinated the participants, analyzed the data, and wrote the manuscript. EB contributed to the design of the study, supervised the acoustic analyses, and reviewed the manuscript. DB conducted the statistical analysis and reviewed the manuscript. GB provided resources for experiments and reviewed the manuscript. DO designed the study, analyzed the data, and wrote the manuscript.

## Conflict of Interest Statement

The authors declare that the research was conducted in the absence of any commercial or financial relationships that could be construed as a potential conflict of interest.

## Supplementary Material

The file labeled Supplementary Table 1 consists of the Appendices. The file labeled Supplementary Data Sheet 1 provides wave files of example utterances and Supplementary Data Sheet 2 provides wave files of the 42 stimuli.
