# Supplementary material for: Acoustic Correlates and Adult Perceptions of Distress in Infant Speech-Like Vocalizations and Cries
Source: Front Psychol. 2019 May 29;10:1154. doi: 10.3389/fpsyg.2019.01154 (PMC6548812; doi:10.3389/fpsyg.2019.01154)
Supplement: Supplementary file 1 [file Table_1.pdf]

Supplementary Material to

Acoustic correlates and adult perceptions of distress in infant speech-like vocalizations and cries

Hyunjoo Yoo, Eugene H. Buder, Dale D. Bowman,

Gavin M. Bidelman, and D. Kimbrough Oller

Appendix 1: Acoustic exemplars

(a) Wail

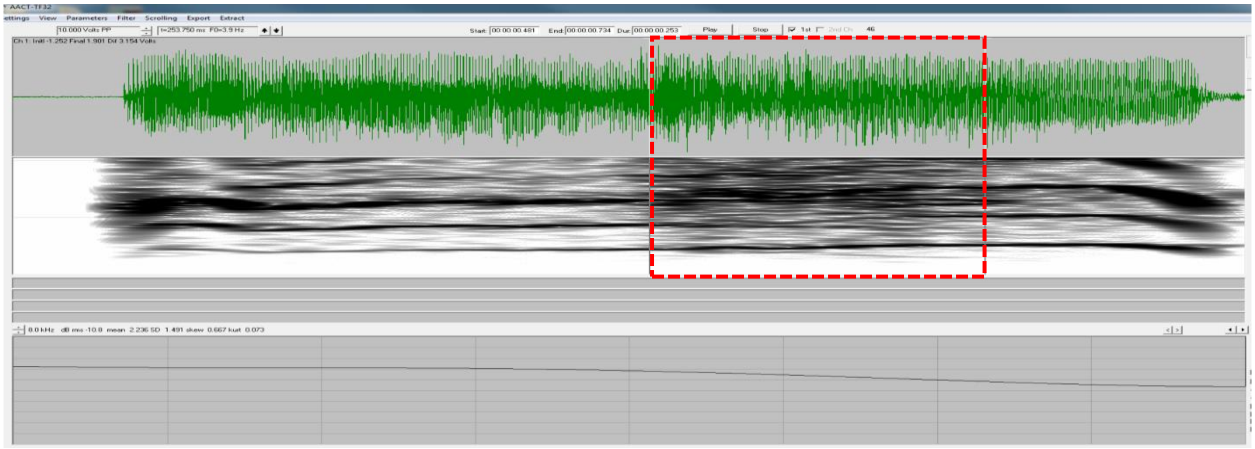

TF32 displays a waveform at the top, a type 2 spectrogram in the middle (2 kHz range), and a long-term spectral average for the period between the cursors (in this case the whole utterance) with 8 kHz range. This wail cry from 0-months shows three regime segments, the middle one ("Aperiodic") being indicated by the red box (dashed line), with the two surrounding segments being Modal. The middle segment provides the most salient high distress information, and its long-term spectral average (~ 2.2 kHz), displayed in the bottom panel, is much higher than in typical vocants.

(b) Whine

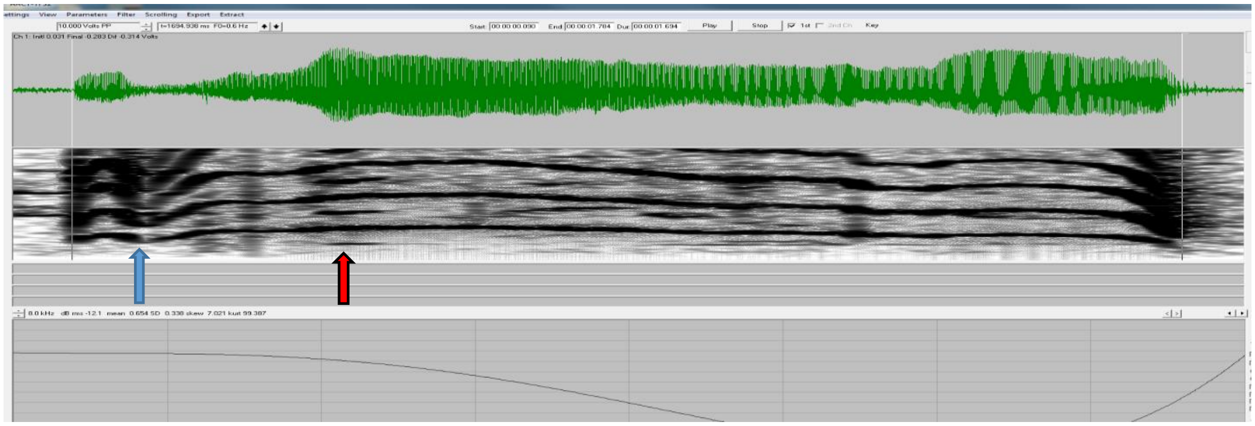

This whine shows more spectral variation than typical vocants, and the whole utterance was categorized as pertaining to the Modal regime. At the beginning of the utterance there is a brief phonatory break (blue arrow: first one) that was counted as an instantaneous break, i.e. a regime. Shortly thereafter a brief subharmonic segment occurs (red arrow: second one), but it was ignored in the coding because of its brevity. The long-term average spectral concentration (~.65 kHz) is intermediate between typical vocants and wails.

(c) Whimper

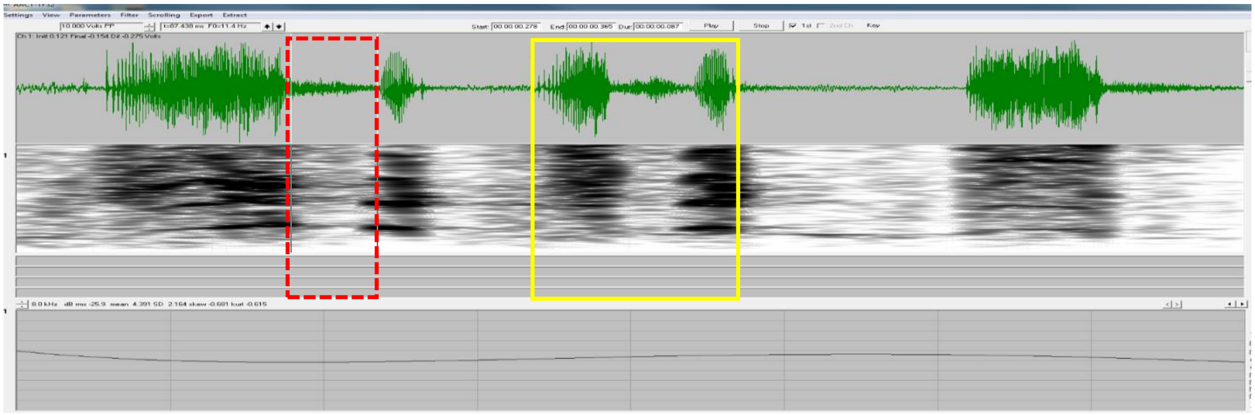

Whimpers are defined to include at least one glottal burst preceded or followed by a short nucleus that is usually somewhat nasalized. Such sequences are unambiguously heard as distressful. Often Whimpers occur in complicated sequences of events as in the example utterance above, a single breath group, including two Whimpers and adjacent whiny sounds. The red box (dashed line) on the left encloses a voiceless glottal burst (~80 ms) which precedes a short nasalized nucleus (~40 ms); that sequence by itself would have constituted Whimper if it occurred in isolation. There is an additional sequence of glottal burst and short nucleus in the utterance to the right (yellow box: solid line), which also would constitute Whimper in isolation. The additional segments are typical possible adjuncts within a Whimper utterance, whiny or voiceless nuclei.

To simplify our comparisons, we did not include Whimpers among the selected stimuli for the present study. The long-term average spectral concentration of the voiceless burst in the first red box was ~4 kHz and in the nasalized nucleus thereafter ~1.3 kHz.

(d) Wail with catch breath

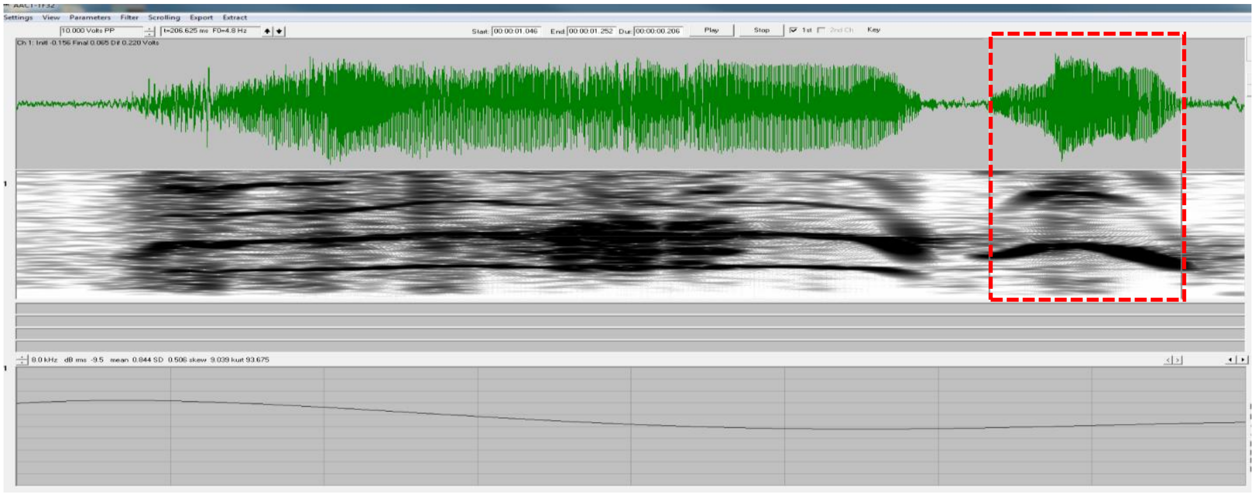

Another complicating factor in cry is the catch breath (in the red box: dashed line), a distinctive marker for cry, defined as an abrupt inspiratory phonation that can (but often does not) occur at the end of high-distress wail. The catch breath seems spasmodic, as if the infant has used up the vital capacity with the egress and is required to inhale rapidly. To simplify our comparisons, we did not include catch breaths among the stimuli for the present study.

(e) Vocant

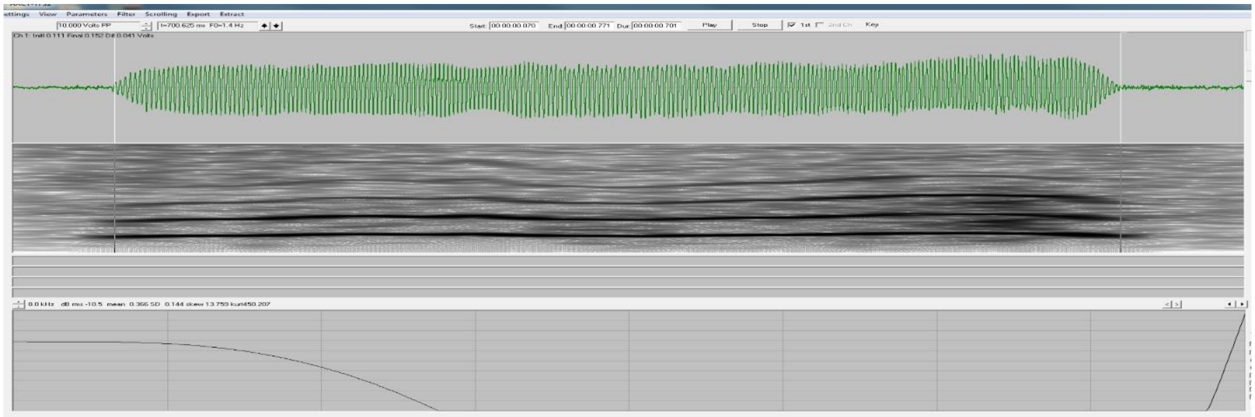

This vocant from a 0-month-old human infant shows a single vibratory regime (Modal), with relatively evenly-spaced and easily-recognized harmonics throughout. The long-term spectral average is low in this utterance (~.36 kHz), reflecting the fact the bulk of the energy is concentrated at low frequencies.

(f) Supraglottal Articulation : multisyllabic utterances

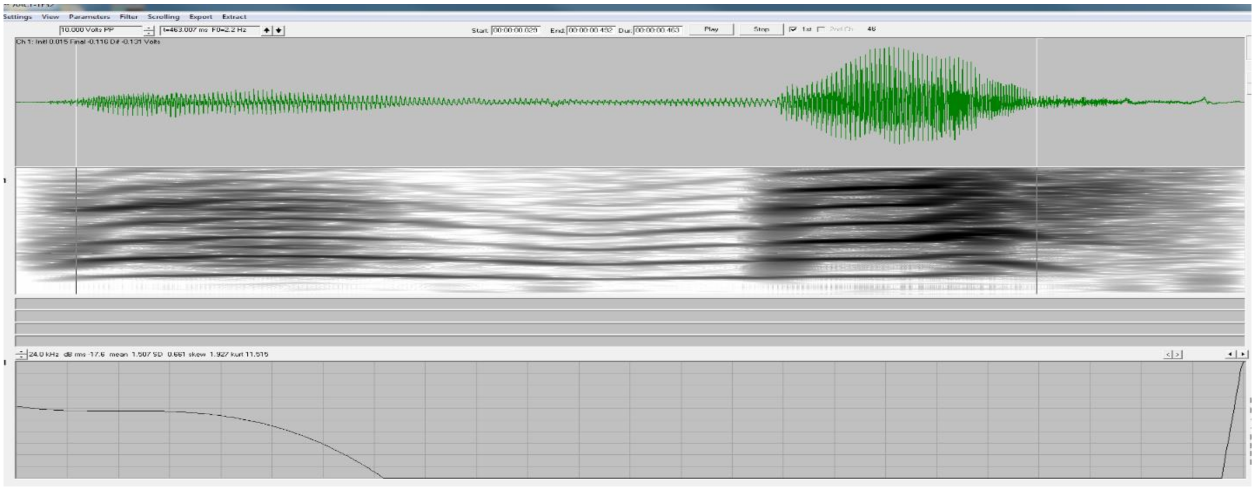

Infant vocalizations can include supraglottal articulation interrupting the phonatory pattern(s). Here a multisyllabic vocant sequence [ama] is displayed. To simplify our comparisons, we did not include utterances in any of the categories (wail, whine, or vocant) if there were syllabifying supraglottal articulations.

Appendix 2: Acoustic parameter selection

We began by evaluating 43 possible parameters signaling vocal distress based on our own prior work (e.g., Yoo, Bowman, & Oller, 2018; Yoo, Buder, Lee, & Oller, 2015; Oller et al. 2013) and that of other researchers (e.g., Green et al., 1987; Gustafson & Green, 1989; Leger, Thompson, Merritt, & Benz, 1996; Fuamenya et al. 2015). We evaluated Pearson correlations between each possible predictor measured in TF32 (Milenkovic, 2018) and the mean ratings of the 39 listeners on the 42 utterances. The correlations provided a basis for culling down the predictors of vocal distress to a relatively small number to submit to multiple regression.

In Table 1 (see below) we present the original set of 17 acoustic measurement types that were subjected to evaluation in several ways, resulting in the 43 parameters for our initial analysis. 8 parameters (mean, max, min, and sd of f_0 and RMS) were evaluated across entire utterances (the *unweighted* method), but we also evaluated 15 of them (all but Duration and Number of Regimes, which was incompatible with the weighted method) to account for their contributions within each regime segment (the *weighted* method). For example, if an utterance consisted of two regimes such as modal and pulse, f_0 was measured twice, once within each regime. After obtaining two f_0 values, a weighted f_0 would be calculated by multiplying each f_0 by the durational proportion of each regime in the utterance and adding those two values.

Furthermore, some of the parameters were evaluated by considering the maximum and/or minimum values across all the regime segments within an utterance (the *segment-specific* method). This approach was evaluated for all the parameters except Duration and Number of Regimes. If there were multiple regimes within an utterance, parameters were measured within each regime.

Correlations between mean distress ratings and the original 43 parameters were evaluated. After obtaining correlations for the 43 parameters (8 unweighted + 15 weighted + 18 segment-specific maxima or minima + Duration + number of regimes), we selected 9 parameters showing the highest correlations within each parameter group, where the groupings represented conceptual association (i.e., they appeared to measure the same essential acoustic properties). These 9 parameters were included in the full model to test for best predictors of the distress ratings.

Table 1: The original set of 17 acoustic measurement types

No.	Parameter	Description
1	Duration	Duration was measured from the onset to the offset of each utterance by placing cursors in TF32, using waveform displays primarily and not including breathy offsets to utterances. TF32 returns a ms accurate duration value.
2-5	Pitch (Fundamental frequency (f_0) mean, max, min, and sd)	f_0 was measured by determining in kHz the frequency of the first harmonic of each utterance. TF32 adapted for AACT traces f_0 using an automated algorithm (autocorrelation) and provides mean, sd, min, and max of f_0. In cases where the algorithms failed to trace f_0 accurately, the first author corrected the f_0 trace using special facilities of TF32. For example, if the trace disappeared or showed values that were transparently incorrect, we adjusted up to six parameters (e.g., the minimum correlation threshold) to invoke a more appropriate tracing, and if the trace remained inappropriate, we manually modified it to the correct values.
6-9	Amplitude (Root-mean-square amplitude, RMS, mean, max, min, and sd)	RMS was used to determine average energy in volts of each utterance, and each segment as provided automatically by TF32 in AACT. RMS was measured at each segment in cases where variations in regime occurred, and weighted values were obtained as appropriate.
10-11	Spectral Ratio (Low- versus high spectral energy ratio, L/H)	This factor has also been shown to help explain dysphonation in speech (Awan, Roy & Dromey, 2009; Hillenbrand & Houde, 1996; Awan, Watts & Awan, 2011). A ratio (in dB) was obtained with two different boundary frequencies (i.e., 4 kHz and 2 kHz). Thus we calculated the ratio between the average energy below 4 kHz (or 2 kHz) and the energy above 4 kHz (or 2 kHz).
12-15	Spectral Mean & Spectral Dispersion (SD) (Spectral moments of the long-term average spectrum, LTAS, mean and sd)	The first and second spectral moments (mean and sd) are useful in obtaining overall spectral shape instead of focusing on fine structure (Forrest, Weismer, Milenkovic, & Dougall, 1988). By selecting mmT in TF32, and turning off pre-emphasis, spectrum plots and moment values for a selected period were generated in the 0-8 kHz frequency range. In the present study, we measured both spectral moments with LTA and without LTA. Mean and standard deviation of spectral moments were measured at each regime segment. After obtaining spectral moment values for each regime segment, weighted values were calculated to adjust for the proportion of each regime type occurring within an utterance.
16	Periodicity (Cepstral peak prominence, CPP)	CPP has been known to be a useful measure for periodicity, particularly in dysphonic speech (e.g., Heman-Ackah et al., 2003). In order to measure CPP in TF32 using LENA recordings (sampling rate 16 kHz), a special updated version of TF32 was developed by Milenkovic. CPP was measured at a typical point of periodicity in each regime segment. The values in dB (high values representing greater dominance of harmonics) were computed without high frequency pre-emphasis in order to maximize comparability with commercially available cepstral analysis tools (Awan, 2011)
17	Number of Regime segments	The number of regime segments designated within the utterance was simply counted (= number of shifts plus one or number of regime tokens, not types)

Appendix 3: Intra-rater and inter-rater statistical comparisons on the influence of acoustic parameters on judgments of distress

a) The Cox-Stuart (CS) test was used to assess intra-rater differences in using acoustic parameters for distress ratings across 10 trials:

This analysis produced a matrix of 9 (acoustic parameters) by 39 (listeners) that contained the p-values of the corresponding CS-test for trend on each acoustic parameter. The null hypothesis of the CS-test for trend for a rater on an acoustic parameter was that there was no trend, and the alternative was that there was a monotonic trend (in either direction) for that rater on that parameter. Lower *p*-values indicate reliable variation in listeners' judgments across trial blocks with regard to that acoustic parameter (see main text Table 4). We then determined the proportion of listeners who differed from chance by the CS-test on each parameter. Finally, we conducted a chi-square test on the proportion of listeners differing from chance for each parameter, thus developing a statistical measure of the extent to which listeners varied their utilization of each parameter across the 10 trial blocks of the task.

b) The permutation procedure and associated tests to assess possible inter-rater differences in use of the various acoustic parameters to make distress judgments involved the following ten steps:

1) We assigned each rater a number from 1 to 39.
2) We selected an acoustic parameter (e.g., Duration).
3) We selected a random integer (n) between 2 and 38 as a basis for forming two subgroups, and then drew a random sample of size n from the integers 1 through 39, corresponding to the n listeners who had been assigned to the randomly selected integers.
4) We split the 39 correlations between ratings and parameters into two groups, one corresponding to the n randomly selected listeners and the other corresponding to the remaining (39 - n) listeners. For example, suppose n = 2 (randomly chosen with equal probability from 2 to 38) on the first permutation trial, T. Then we would choose a sample of size 2 from the integers 1 through 39, say 8 and 17, corresponding to listeners 8 and 17. Then we would split the correlations of the 39 listeners into a sample containing the correlations of the acoustic parameter (e.g., Duration) with the distress ratings of the 8th and 17th listeners and another sample containing the correlations of all the listeners except the 8th and the 17th (i.e., the 37 other listeners). Once two subgroups were established:
5) We conducted a non-parametric test (Kolmogorov-Smirnov) to determine the likelihood that the two groups of correlations came from the same population, and we recorded the resulting *p*-value.
6) ~ 10,000 such trials, T, were conducted for each acoustic parameter; if T < 10,000, we returned to 3) above for the next permutation trial on the acoustic parameter (e.g., Duration), which would begin with selection of another random integer between 2 and 38, say 14. Then another step 4) would select a random sample of size 14 from among the listeners, and the correlations of their ratings with the selected acoustic parameter at step 5) would be compared by the Kolmogorov-Smirnov test against the correlations of those of the other 25 (39-14 = 25) listeners. The probability that these samples had been drawn

from the same population would then be recorded, and if T < 10,000, the next permutation trial would begin again at step 3.

7) After ~ 10,000 randomly selected pairings of groups of listeners' correlations for an acoustic parameter had been compared by the Kolmogorov-Smirnov test, we tabulated all the p-value results for that parameter (e.g., Duration) indicating the likelihood that the pairs (the two groups of correlations of ratings) had been drawn from the same population, and repeated the entire procedure from step 3) through 5) for the next parameter (e.g., Average Pitch (f_0)). After all nine acoustic parameters had been tested:

8) We computed the proportion of the ~ 10,000 pairings where the Kolmogorov-Smirnov tests for each acoustic parameter failed to reject the null hypothesis that the pairs were from the same population at $p < .05$, that is, that their correlations were not more different than would be expected by chance.

9) To determine whether inter-rater variation was greater than chance on any acoustic parameter, we tested the observed proportion of 10,000 trials rejecting the null versus chance, using a chi-square test. For example, if 15% of 10,000 pairings differed from chance at $p < .05$ based on the Kolmogorov-Smirnov test on an acoustic parameter (e.g., Duration) then a two-by-two chi-square test would compare chance (500 rejections compared to 9500 failures to reject at $p < .05$) against the obtained number of permutation trials where, in accord with the .05 criterion, the null hypothesis for Duration had been rejected (1500 compared to 8500) and would determine that the chi-square difference from chance was highly significant ($p < .00001$). We could then conclude that listeners differed significantly from each other (showed significant inter-rater variation) in the correlations of their ratings with the parameter Duration.

10) To compare the inter-rater variation between two different acoustic parameters, we compared parameters that did not show significantly more trials rejecting the null hypothesis at $p < 0.05$ than would be expected by chance with parameters that did show more significant differences than would be expected by chance. A chi-square test was used to test for significant differences between the two parameters, say Duration and Average Pitch (f_0). For example if only 550 of the 10,000 trials rejected the null hypothesis on Average Pitch (f_0), we could test Duration (1500 compared to 8500) against Average Pitch (f_0) (550 compared to 9450) and determine that the acoustic parameter Average Pitch (f_0) showed significantly ($p < .00001$) *less* inter-rater variation than the parameter Duration.

Appendix 4: The Role of Experience in Coding on Distress Ratings

Acoustic Parameter	*p*-value for Wilcoxon test, experienced vs inexperienced listeners
Duration	0.79
Average Pitch (f_0)	0.65
Max Pitch (f_0)	0.18
Max Amplitude (RMS)	1.0
Spectral Ratio	**0.0001**
Spectral Mean	**0.02**
Spectral Dispersion (SD)	**0.0006**
Periodicity	0.09
Number of Regimes	0.38

Appendix 5. The relationship between Duration and Number of Regimes

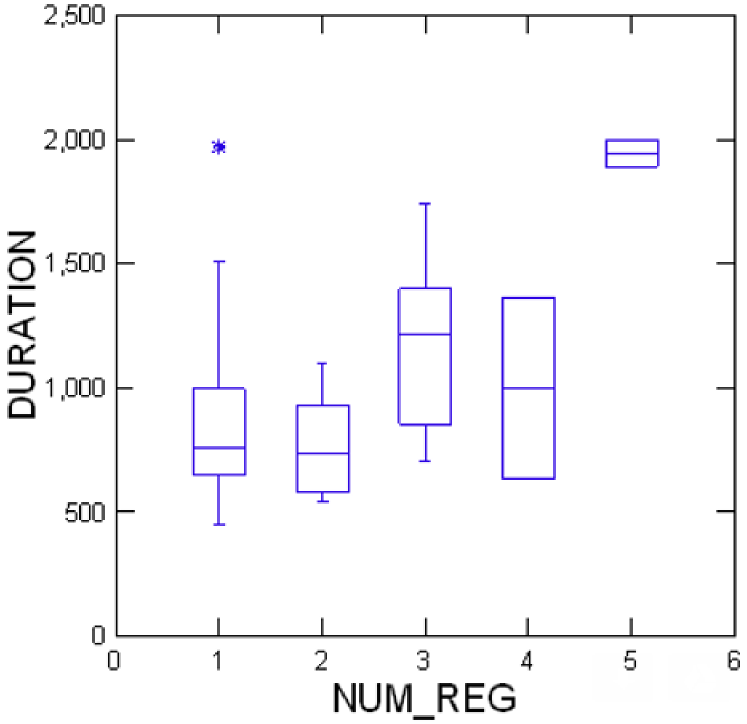

Fig. The relationship between Duration and Number of Regimes.

The box plot shows that utterances with 1-4 regimes spanned quite similar ranges of Duration and only utterances with 5 regime segments appeared notably longer. There were two cases of outliers showing quite long duration but containing only 1 regime (displayed with the * symbol in the figure).

Table 1. ANOVA comparisons of Duration and Number of Regimes

Source	Type III SS	df	Mean Squares	F-ratio	*p*-values
Num-Reg	2726171.656	4	681542.914	4.498	0.005
Error	5606840.629	37	151536.233		

Table 2. Post hoc comparisons using the Tukey HSD test

Num_Reg (i)	Num_Reg (j)	Difference	p-value	95% Confidence interval	
				Lower	Upper
1	2	144.8	0.918	-352.3	642
1	3	-277	0.308	-683.2	129.2
1	4	-80.9	0.999	-912.7	750.9
1	5	-1029.4	0.009	-1861.2	-197.6
2	3	-421.8	0.164	-945	101.4
2	4	-225.7	0.950	-1120.5	669.1
2	5	-1174.2	0.005	-2069	-279.4
3	4	196.1	0.963	-651.6	1043.7
3	5	-752.4	0.102	-1600.1	95.2
4	5	-948.5	0.128	-2064.5	167.5

The correlation between Duration and Number of Regimes variables was r = .44. However, as reported in the manuscript, the Number of Regimes variable explained unique variance in distress level judgments above and beyond that explained by the Duration variable, verifying that the variables were not redundant.

A one-way ANOVA with Number of Regimes as a five-level IV and Duration as the DV was significant (see Tables 1 and 2 above), yet Tukey HSD post hoc tests for pairwise differences revealed that only two comparisons accounted for this result: 1 vs. 5 regime segments and 2 vs. 5 regime segments. In fact, only 2 utterances (out of 42) had 5 regime segments, and there were notable exceptions to this relationship between variables, such as quite long duration vocalizations containing only 1 regime (these are displayed with the * symbol in the figure).

Taken together, these analyses revealed that although there was an overall relationship between Number of Regimes and Duration, the results were driven just 2 out of the 42 cases and there were notable exceptions showing the opposite pattern.

Furthermore, as we briefly mentioned in discussion, Duration was the only variable that predicted the level of distress in the full model of multiple regression. After we incorporated the regime-specific approach, we eventually found that three other variables (Average Pitch, Spectral Ratio, and Number of Regimes) were also strong predictors. In sum, duration of utterances may influence perception of distress levels. However, our study suggests that the regime-specific approach seems to better predict the distress level perception.

Appendix 6. Thoughts on the origin of human communication in general, vocal language in particular, and the expression of distress

Our study illustrates that human listeners come well-prepared to judge vocalizations of human newborns as being either speech-like, cry-like, or in between. Such a capability is surely relevant to the intuitive parenting task of engaging infants in protoconversation (with protophones) while treating whines and cries as signals of need (Yoo et al., 2018). Foundational interactions between parents and infants focused on precursors to speech can only be established if parents recognize and respond differentially to the material of potential speech in infant sounds.

The significance of our findings can be placed in perspective by considering how natural selection could have yielded patterns of vocalization in human infants that express needs as well as well-being, along with patterns of perception in human caregivers that lead to the complex communication system we witness, a system allowing complex expressions of distress alongside an even more complex system of vocal expression in language. In protophones, as in language, distress can be expressed, but in fact typically is not. To understand how we think this multifarious system of communication arose in hominin evolution, consider the logic of our reasoning:

1) We presume that, as with other mammals, hominins have long experienced natural selection on the capacity to produce vocal distress signals, and we presume the capacity to produce wail cry in human infancy is a product of that selection (Bard, 2000; Darwin, 1872; Kojima, 2003; Soltis, 2004).

2) We also presume that hominin *infants* have experienced natural selection on the capacity and the inclination to produce vocalizations (we call them protophones, vocants being the most frequent type of protophone) that are not required to show distress at all, nor to express any other emotional state, although they can express any emotional state on individual occasions of use (Oller et al. 2013). The infant protophones are usually produced in exploratory, playful activities, seemingly devoid of any emotion other than interest in the activity itself. Oller & Griebel (2005) and Locke, (2006) have hypothesized that the selection pressure for infant flexible vocalization was the result of altriciality of the hominin infant, whose protophone production was selected as a fitness signal to caregivers, who presumably invested most in the well-being of hominin infants that most effectively showed their fitness throughout the long helpless period of hominin infancy and childhood. Similarly it is proposed that hominin *caregivers* have long been under selection pressure to *recognize* fitness signals in the form of flexible infant vocalizations—the current results offer support for that proposal, since it is shown here that human listeners (potential caregivers) have a substantial capacity to recognize vocalizations as expressing variable levels of distress.

3) The flexible vocal capacity of the human infant, in recent evolutionary time, forms a foundation for language, where vocal expression is usually not distressful, and where in fact any word or sentence can be produced at will, that is in any circumstance of emotion or lack of it. Thus protophones can be, and usually are, produced by the human infant in circumstances lacking distress (Oller, 2000).

4) While wail cry is necessarily distressful, we do not assume that it is without gradation. There are intense wail cries and less intense ones, a pattern of gradedness that is very common in mammal vocalizations (Marler, 1976; Ploog, 1992; Winter, Ploog, & Latta, 1966).

5) Similarly, while most protophones are produced with no discernible distress, they can accompany varying levels of distress. Thus a continuum from wail cry to vocants can be envisioned theoretically (and our experience suggests it occurs naturally in human infants), even though we presume there have been different selection pressures involved in the evolution of the capacity and inclination to produce wail cries and vocants, one pressure on distress expression and another pressure on flexibility of expression, the value of which initially was in fitness signaling and later in evolution in providing a foundation of more language-like communication.

6) In addition we think it likely that the circumstances that inspire production of distressful sounds in infancy and the circumstances that inspire the production of vocants should not be viewed as entirely separate. It appears to us, based on observational research, that these circumstances can occur simultaneously, intertwined, yielding a mixture of vocalization qualities corresponding to distress and/or lack of it. Our study is an empirical evaluation of the nature of distress expression across the resulting continuum.

These thoughts reflect our goal of illuminating the foundations of language. So while we do not view vocants as words or sentences of language, we do view them as manifestations of an emerging capacity to produce flexible vocalization, a kind of expression not motivated by distress. Such a flexible vocal capacity is a clear requirement of language, and thus while protophones are not words, they can reasonably be viewed as precursors to words (Koopmans-van Beinum & van der Stelt, 1986; Stoel-Gammon & Cooper, 1984). We are also studying the possibility that such flexible vocalization (in addition to distress vocalization) is present in infants of our nearest phylogenetic relatives. The results from our study of bonobo mother-infant pairs (Oller et al., 2019) suggests that vocant-like utterances may indeed occur, although at very much lower rates than in human mother-infant pairs. In various ways our research is exploring language-like vocalization in infancy as an especially human activity, but we are continually drawn to consider language-like vocalization in relation to all its possible functions, including of course the expression of distress.

Appendix 7. Are human cries really confined to expressions of distress?

One might ask if human cry is really entirely an expression of distress. In fact in adulthood, crying seems often to be an expression of relief or even joy. Green and colleagues (personal communication and (Green, Whitney, & Potegal, 2011) have also claimed that the human infant cry separates into two forms around 6 months of age, from being merely a distress signal in the first half year to becoming one type for pure distress and another for anger in the second half year. Our present study however pertains to vocalizations of the first three months, prior to the split. Of course anger can also be viewed as negative and perhaps distressful; nonetheless, it does seem likely that the human infant's control of vocalization diversifies by the second-half year, and that by then even cry involves some flexibility of expression.

A similar point has been made with regard to non-human sounds that are often thought of as having fixed expression limits. Sea gull calls, for example, are sometimes produced in apparent distress circumstances, while other times the calls seem to show aggression, suggesting some level of flexibility. The presumed fixed functions of animal vocalizations have also been explored in studies of the presumed "alarm" vocalizations in a variety of species, including primates (Price et al., 2015). The famous vervet alarm calls are in fact produced both in circumstances of predator alarm and in circumstances of intra-specific aggression, a fact that fundamentally undercuts the interpretation of the calls as "predator specific" (Price, 2013; Seyfarth, Cheney, & Marler, 1980). In fact, like human infant cry and sea gull calls, the pattern of usage actually suggests the calls may represent somewhat diversified functions expressing varying kinds of or mixtures of distress, aggression, and fear.
